# Seasonal Phytochemical Variation and Bioactivity of *Artemisia herba‐alba*: Comparative Evaluation of Essential Oils and Phenolic Extracts With Antioxidant, Tyrosinase Inhibitory and In Silico Studies

**DOI:** 10.1002/cbdv.71777

**Published:** 2026-09-27

**Authors:** Alia Djekidel, Boulanouar Bakchiche, Hadjira Guenane, Imededdine Kadi, Hicham Gouzi, Ilyas Yildiz, Süleyman M. Çelik, Ramazan Erenler, Mosad A. Ghareeb, Stefania Garzoli

**Affiliations:** ^1^ Laboratory of Biological and Agronomic Sciences (LBAS) Amar Telidji University Laghouat Algeria; ^2^ Research Unit on Medicinal Plants Biotechnology Research Center Laghouat Algeria; ^3^ Department of Molecular Biology and Genetics Tokat Gaziosmanpasa University Tokat Turkey; ^4^ Department of Medical Services and Techniques, School of Health Services Igdir University Igdir Turkey; ^5^ Department of Chemistry, Faculty of Arts and Sciences Tokat Gaziosmanpasa University Tokat Turkey; ^6^ Medicinal Chemistry Department Theodor Bilharz Research Institute Giza Egypt; ^7^ Department of Chemistry and Technologies of Drug Sapienza University Rome Italy

**Keywords:** antioxidant, *Artemisia herba‐alba*, essential oils, health promotion, in silico, phenolics, seasonal variation, tyrosinase inhibitory

## Abstract

This study estimated the effect of seasonal variation on the chemical composition and biological activities of *Artemisia herba‐alba* hydroalcoholic extract and essential oil, focusing on antioxidant and tyrosinase inhibitory activities. Chemical profiling was achieved using LC–MS for the extract and GC–MS for the essential oil. Total phenolic and flavonoid contents were estimated by the Folin–Ciocalteu's and aluminium chloride colorimetric assays. Antioxidant activity was evaluated using DPPH, ABTS, phosphomolybdate, and FRAP methods, while tyrosinase inhibition was expressed as IC_50_ values using kojic acid as a positive control. Molecular docking was performed to explore the interactions of the main constituents with the tyrosinase enzyme.

LC–MS analysis identified naringenin, vanillin, luteolin, quercetin, and trans‐cinnamic acid as the major compounds across all samples. GC–MS disclosed seasonal variation in essential oil composition; autumn oil contained several unique ingredients, remarkably davana ether 2, while winter oil was characterized by high carvacrol content. The winter essential oil and summer extract exhibited the strongest antioxidant and tyrosinase inhibitory activities. Molecular docking revealed strong binding affinities of vanillin, kojic acid, and carvacrol within the catalytic pocket of tyrosinase, while thujone isomers exhibited weaker but consistent interactions.

## Graphical abstract

*Artemisia herba‐alba*: Phytochemistry and bioactivity of essential oils and phenolic extracts.

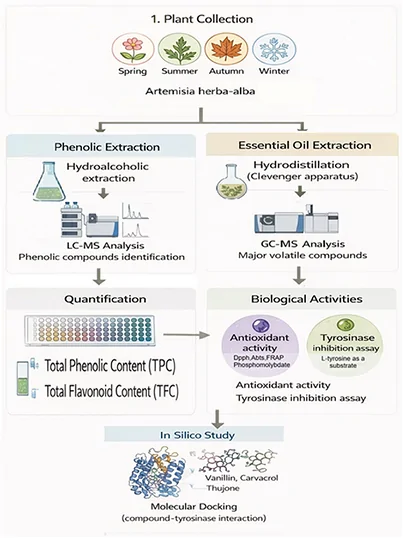

**Abbreviations**
3D: three‐dimensional
ABTS: 2,2′‐azinobis (3‐ethylbenzothiazoline‐6‐sulfonic acid) radical cation.
ACTH: adrenocorticotropic hormone
Akt: protein kinase B
ALLSKY_SFC_SW_DWN: all‐sky surface shortwave downward irradiance
ANOVA: analysis of variance
ARE: antioxidant response element
cAMP: cyclic adenosine monophosphate
CE: collision energy
CREB: cAMP response element‐binding protein
CRediT: contributor roles taxonomy
DAD: diode array detector
DMSO: dimethyl sulfoxide
DOPA: 3,4‐dihydroxyphenylalanine.
DPPH: 2,2‐diphenyl‐1‐picrylhydrazyl radical.
dw / d.w.: dry weight
EIMS: electron impact mass spectrometry
EO: essential oil
ESI: electrospray ionization
ET‐1: endothelin‐1
FRAP: ferric reducing antioxidant power
GAE: gallic acid equivalents
GC–MS: gas chromatography–mass spectrometry
GWETTOP: surface soil wetness
HPLC: high‐performance liquid chromatography
IC_50_
: half‐maximal inhibitory concentration
LBSA: Laboratory of Biological and Agronomic Sciences
LC–MS: liquid chromatography–mass spectrometry
LC–MS/MS: liquid chromatography–tandem mass spectrometry
L‐DOPA: L‐3,4‐dihydroxyphenylalanine
LOD: limit of detection
LOQ: limit of quantitation
*m/z*
: mass‐to‐charge ratio
MC1R: melanocortin 1 receptor
MH: monoterpene hydrocarbons
MITF: microphthalmia‐associated transcription factor
MRM: multiple reaction monitoring
NASA POWER: NASA Prediction of Worldwide Energy Resources
NIST: National Institute of Standards and Technology.
Nrf2: nuclear factor erythroid 2‐related factor 2
OM: oxygenated monoterpenes
OS: oxygenated sesquiterpenes
PDB: Protein Data Bank
PI3K: phosphoinositide 3‐kinase
PM: phosphomolybdenum assay
QE: quercetin equivalents
RCSB: Research Collaboratory for Structural Bioinformatics.
RE: rutin equivalents
RH2M: relative humidity at 2 meters
RI: retention index
RICal: calculated retention index
RILit: literature retention index
ROS: reactive oxygen species
RUMP: Research Unit of Medicinal Plants
SDF: structure data file
SDS: sodium dodecyl sulfate
SEM: standard error of the mean
SH: sesquiterpene hydrocarbons
SPSS: Statistical Package for the Social Sciences
T2M: temperature at 2 meters
TFC: total flavonoid content
TIC: total ion chromatogram
TPC: total phenolic content
VCEAC: vitamin C equivalent antioxidant capacity
WS2M: wind speed at 2 meters
α‐MSH: alpha‐melanocyte‐stimulating hormone
ΔG: Gibbs free energy

## 1 Introduction

*Artemisia herba‐alba* is a greenish‐silver, perennial, strongly aromatic dwarf shrub that grows wild in arid areas of the Mediterranean basin extending into the northwestern Himalayas. This plant, commonly known as the white wormwood in Arabic as “*Chih*” and in France as “Armoise blanche”, is used in folk medicine. It was known for its therapeutic and medicinal properties and was used for the treatment of colds, coughing, and intestinal disturbances; as an antidiabetic agent; for bronchitis, diarrhoea, neuralgias, and hypertension. Many researchers have reported various biological and/or pharmacological activities of *A. herba‐alba* essential oil as an antibacterial (bacteria and fungi), antileishmanial, anthelmintic, and antispasmodic agent [1].

This plant derives its therapeutic value mainly from two classes of secondary metabolites. Its essential oil appears to be characterized by a dominance of oxygenated monoterpenes: camphor (17%–55%), *α*‐thujone (7%–48%), *β*‐thujone, chrysanthenone, 1,8‐cineole, and borneol, with marked variability across Algerian chemotypes [2, 3, 4, 5]. Its phenolic fraction is rich in phenolic acids (caffeic, *p*‐coumaric, ferulic, gallic, cinnamic, and protocatechuic acids) and flavonoids (apigenin, luteolin), which together are responsible for the strong radical‐scavenging capacity [6, 7, 8]. The biosynthesis of these metabolites is highly dependent on seasonal factors, with documented variations in essential oil production (0.2%–0.9% v/d.w.) and phenolic load at different phenological stages [2, 9, 10, 11]. These changes directly affect the antioxidant and enzyme‐inhibiting bioactivity of the plant [6, 8, 12].

In addition to its antioxidant activity, components of *A. herba‐alba* may exhibit tyrosinase‐inhibitory activity. This enzyme catalyzes the oxidation of L‐tyrosine to L‐DOPA and dopaquinone, with the production of superoxide and hydrogen peroxide, and is involved in several hyperpigmentation disorders including melasma, post‐inflammatory hyperpigmentation, and solar freckles [13, 14, 15]. Melanin production is closely related to oxidative stress and the Nrf2‐ARE pathway, where down‐regulation of Nrf2 expression causes an increase in the activity of melanocytes and tyrosinase, and the activation of Nrf2 reduces the expression of tyrosinase through the PI3K/Akt pathway [15, 16, 17, 18]. Other wormwood species back this dual‐angle approach. Huang et al. reported that *A. argyi* essential oil inhibited mushroom tyrosinase (IC_50_ = 19.16 mg/mL) [19], and Pereira et al. found IC_50_ values as low as 4.13 mg/mL for phenolic‐rich extracts of *A. campestris* subsp. Maritima [20], suggesting the metabolic repertoire of *A. herba‐alba* may contribute to its ability to inhibit melanin production [14, 21, 22, 23].

In plants, tyrosinase inhibition is mostly attributed to polyphenols and flavonoids but the essential oil component has been given far less attention, with a recent survey showing that only a few of the 71 oils studied showed significant tyrosinase‐inhibiting activity [24]. The major constituents of *A*. *herba‐alba* essential oil have been reported to possess tyrosinase inhibition activities in previous studies. For instance, 1,8‐cineole seems to interact with the active site of the mushroom tyrosinase enzyme [25], and thymol was found to be the major anti‐melanogenic element in a related wood essential oil [26]. Nevertheless, despite the reported specific pharmacological activities of *A. herba‐alba*, there is a significant dearth of integrated seasonal metabolomics, tyrosinase inhibition, and molecular docking studies. The effects of seasonal dynamics on the chemical composition and enzyme target binding are essential for determining the best harvesting methods. Accordingly, this study was undertaken to assess the seasonal variations in the composition of essential oils and phenolic compounds of *A*. *herba‐alba*, its antioxidant and tyrosinase inhibition potential, and to conduct a computational analysis of the binding of major metabolites to tyrosinase to identify the best harvesting time of this species for pharmaceutical and cosmetic uses. Moreover, the present work supports Sustainable Development Goal 3 (Good Health and Well‐being) by advancing drug discovery efforts through the investigation of natural product‐derived compounds with potential therapeutic applications against various diseases.

## 2 Materials and Methods

### 2.1 Chemicals and Reagents

The chemicals DPPH (2,2‐diphenyl‐1‐picrylhydrazyl radical), ABTS (2,2′‐azinobis‐(3‐ethylbenzothiazoline‐6‐sulfonic acid) radical cation), and Folin–Ciocalteu phenol reagent were purchased from Sigma‐Aldrich (St. Louis, MO, USA). BHT (butylated hydroxytoluene), gallic acid, ascorbic acid, rutin, Trolox, L‐tyrosine, and kojic acid were purchased from Aldrich Chemical Co. (USA). All other chemicals were of analytical grade.

### 2.2 Plant Material

Aerial parts (including stems and leaves) of *A. herba‐alba* were gathered from their natural habitat during the four seasons: Spring (March 2023), Summer (July 2023), Autumn (September 2023), and Winter (November 2023) in the Aflou zone, corresponding to the following coordinates: 34°06'50''N, 2°05'50''E, located 100 km from the northwest of Laghouat city, southern Algeria. A randomized sampling protocol was implemented to select healthy, mature specimens at the zenith of their seasonal growth phase, which may minimize variability. Samples were collected in clear morning weather and grouped into representative composite samples for each season to achieve consistent seasonal comparisons. Professor Mohamed Kouidri identified this plant at the Agricultural Department of the Faculty of Sciences at Laghouat University in Algeria. Voucher specimens were deposited at the Laboratory of Biological and Agronomic Sciences (LBSA) Laghouat University Algeria, under the voucher numbers LBSA‐AHA/03/23, LBSA‐AHA/07/23, LBSA‐AHA/09/23, and LBSA‐AHA/11/23, respectively. The plant material was shaded and dried (at room temperature) for 2 weeks, then ground and stored in paper sacks until needed.

### 2.3 Preparation of the Ethanolic Extracts

Ten grams of air‐dried plant material underwent crushing and extraction for 72 h with 100 mL of 80% (v/v) aqueous ethanol at room temperature. The material was filtered, and the crude extract obtained was depigmented by partitioning with *n*‐hexane to remove chlorophyll and other pigments, the solvent was then evaporated using a rotary evaporator, and the dried extracts were weighed. The extraction yield was calculated as the ratio between the dried extract and initial air‐dried plant material in percentage. The extraction yields were 18.7%, 22.3%, 20.1%, and 18.3% for spring, summer, autumn, and winter, respectively. The dried extracts were stored in dark, airtight containers at 10°C until further analysis.

### 2.4 Determination of Total Phenolic Content

The total phenolic content of extracts was determined according to the Folin–Ciocalteu procedure described by Boulanouar et al. (2013) [27] with slight modification. A calibration curve was constructed using gallic acid, and the results were expressed as milligrams of gallic acid equivalent per gram dry weight (DW) of the extract (mg GAE/g DW), which may constitute a standardized measure of phenolic content. All tests were performed in triplicate for each extract.

### 2.5 Determination of Total Flavonoid Content

The total flavonoid content of extracts was determined by the method described by Boulanouar et al. (2013) [27], based on the formation of a flavonoid–aluminium complex exhibiting maximum absorbance at 430 nm. Total flavonoid content was expressed as Rutin equivalent per gram dried weight (mg RE/g DW), a unit that may facilitate inter‐study comparisons. Tests were performed in triplicate for each extract.

### 2.6 Essential Oil Extraction

The essential oil was extracted from the aerial parts of *A. herba alba* using a Clevenger‐type apparatus, a technique widely used for extracting EO while preserving its chemical properties. For this, 100 g of each sample was distilled in distilled water (1 L) for 3 h. Anhydrous sodium sulfate was used for drying the volatile oils. The essential oil yield based on % w/w was determined on dry vegetable matter. The extracted essential oils were maintained in a refrigerator (+4 °C) until further analysis [28], a protocol which may serve to preserve chemical integrity.

### 2.7 Chromatographic Analysis

#### 2.7.1 Liquid Chromatography‐Mass Spectrometry

Sample preparation was performed by weighing 50 mg of dry extract into a 2 mL Eppendorf tube and dissolving it in 1 mL of a solvent mixture consisting of acetonitrile, methanol, and water (1:1:1, v/v/v). The mixture was vortexed, and any remaining insoluble material was completely dissolved using an ultrasonic bath, a process which may enhance extraction efficiency. To remove non‐polar lipid interferences, 0.8 mL of n‐hexane was added to the extract, followed by centrifugation at 7000 rpm for 5 min. The lower aqueous phase was collected, diluted (1:4, v/v), and filtered through a 0.25 (µm/m) syringe filter before injection.

The utilization of a 1260 Infinity HPLC, LC–MS/MS system, coupled with an Agilent 6460 triple quadrupole mass spectrometer (USA), appears appropriate for the experimental requirements. The separation process utilized an Agilent Poroshell 120 EC‐C18 (50 mm × 4.6 mm I.D., 2.7 µm), a type of reversed‐phase column. The LC separation was performed using gradient elution with a mobile phase consisting of water containing 0.1% formic acid and 5 mM ammonium formate (Mobile Phase A) and acetonitrile containing 0.1% formic acid (Mobile Phase B). The gradient elution program was set as follows: 0 min (15% B), 5 min (25% B), 15 min (75% B), 16–20 min (100% B), and 22–40 min (15% B for column re‐equilibration). The injection volume was 4.00 µL, the flow rate was 0.4 mL/min, and the temperature was maintained at 30°C. Mass spectrometric detection was operated in multiple reaction monitoring (MRM) mode using electrospray ionization (ESI) in both positive and negative modes. Target analytes may be identified and quantified based on precursor/product ion transitions, fragmentor voltages, and collision energies, which appear to have been optimized using authentic commercial reference standards (*R* ^2^ ≥ 0.99).

#### 2.7.2 Gas Chromatography–Mass Spectrometry

The hydrodistilled EOs were diluted to 5% in HPLC‐grade *n*‐hexane and then injected into a GC–MS apparatus. Gas chromatography–electron impact mass spectrometry (GC–EIMS) analyses were performed with an Agilent 7890B gas chromatograph (Agilent Technologies Inc., Santa Clara, CA, USA) equipped with an Agilent HP‐5MS (Agilent Technologies Inc., Santa Clara, CA, USA) capillary column (30 m × 0.25 mm; coating thickness 0.25 µm) and an Agilent 5977B single quadrupole mass detector (Agilent Technologies Inc., Santa Clara, CA, USA). Analytical conditions were as follows: injector and transfer line temperatures 220°C and 240°C, respectively; oven temperature programmed from 60°C to 240°C at 3°C/min; carrier gas helium at 1 mL/min; injection of 1 µL (5% HPLC grade n‐hexane solution); split ratio 1:25. Acquisition parameters were as follows: full scan; scan range: 30–300 *m/z*; scan time: 1.0 sec [28]. The identification of the constituents was based on the comparison of their retention times with those of the authentic samples (when available), comparing their linear retention indices relative to the series of *n*‐hydrocarbons (C6–C25). Computer matching was also used against a commercial (National Institute of Standards and Technology 2014) and a laboratory‐developed mass spectra library built up from pure substances and components of commercial essential oils of known composition and MS literature data.

### 2.8 Antioxidant Activity

#### 2.8.1 DPPH Radical‐Scavenging Capacity

The free radical scavenging ability was established according to the method used by Hadjira et al. (2025) [29]. Briefly, a volume of 200 µL of the sample (essential oil or extract diluted in ethanol) at different concentrations ranging from 0 to 1 mg/mL was added to 1800 µL of the DPPH solution (0.1 mM). After 30 min of incubation at room temperature in the obscurity, the absorbance was read at 517 nm using a multimode plate reader. Vitamin C was considered as the antioxidant standard, all measurements were performed in triplicate (*n* = 3). The inhibition percent was calculated as follows:

$$\mathrm{Inhibition}\left(\%\right)=\left[\left(\mathrm{Abs}\;\mathrm{control}-\mathrm{Abs}\;\mathrm{sample}\right)/\mathrm{Abs}\;\mathrm{control}\right]\;\times \;100$$

The results were represented as IC_50_ values (µg/mL), which corresponds to the concentration of 50% inhibition.

#### 2.8.2 ABTS (2,2'‐azinobis (3‐thylbenzothiazoline)6‐ sulfonic) Radical Trapping Activity

The anti‐radical activity of the extract against ABTS^•+^ radical was realized by the method described by Hadjira et al. (2025) [29]. The ABTS^•+^ solution was obtained by combining 7 mM of ABTS in water with 2.45 mM of potassium persulfate, stored in the obscurity at room temperature for 16 h. The ABTS^•+^ solution was diluted in water to an absorbance of 0.7 at 734 nm. 1900 µL of this solution was added to 100 µL of the sample (essential oil or extract diluted in ethanol) at different concentrations ranging from 0 to 1 mg/mL. After 10 min of incubation, the absorbance was recorded at 734 nm, all measurements were performed in triplicate (*n* = 3). The inhibition percent was calculated as follows:

$$\mathrm{Inhibition}\left(\%\right)=\left[\left(\mathrm{Abs}\;\mathrm{control}-\mathrm{Abs}\;\mathrm{sample}\right)/\mathrm{Abs}\;\mathrm{control}\right]\;\times \;100$$

The results were represented as IC_50_ values (µg/mL), which corresponds to the concentration of 50% inhibition.

#### 2.8.3 Phosphomolybdenum Assay

The antioxidant activity of the extract was evaluated by the phosphomolybdenum method described by Hadjira et al. (2025) [29]. The assay is based on the reduction of Mo(VI)–Mo(V) by the extract and subsequent formation of a green phosphate/Mo(V) complex at acid pH. A 0.3 mL of the sample (essential oil or extract diluted in ethanol) at different concentrations ranging from 0 to 1 mg/mL, was combined with 3 mL of reagent solution (0.6 M sulphuric acid, 28 mM sodium phosphate, and 4 mM ammonium molybdate). In case of blank, 0.3 mL of methanol was used in place of extracts. The tubes containing the reaction solution were capped and incubated in a boiling water bath at 95°C for 90 min. After cooling at room temperature, the absorbance of the solution was measured at 695 nm using a spectrophotometer, all measurements were performed in triplicate (*n* = 3), results were expressed as equivalents of ascorbic acid (VCEAC) (mmol/g).

#### 2.8.4 Ferric‐Reducing Antioxidant Power Assay (FRAP)

The reducing power of extracts was determined according to the method previously described by Hadjira et al. (2025) [29]. Briefly, 1 mL of the sample (essential oil or extract diluted in ethanol) at different concentrations ranging from (0 to 1 mg/mL) was mixed with phosphate buffer (2.5 mL, 0.2 M, pH 6.6) and potassium ferricyanide [K_3_Fe(CN)_6_] (2.5 mL, 1%). The mixture was incubated at 50°C for 20 min. A portion (2.5 mL) of trichloroacetic acid (10%) was added to the mixture, which was then centrifuged at 3000 rpm for 10 min. The upper layer of the solution (2.5 mL) was mixed with distilled water (2.5 mL) and FeCl_3_ (0.5 mL, 0.1%) and the absorbance was measured at 700 nm. Increased absorbance of the reaction mixture indicated increased reducing power. Phosphate buffer (pH 6.6) was used as blank solution. All measurements were performed in triplicate (*n* = 3). Results were expressed as equivalents of ascorbic acid (VCEAC) (mmol/g).

### 2.9 Tyrosinase Inhibition

Mushroom tyrosinase was obtained from *Agaricus bisporus* and partially purified by the method described by Gouzi et al. (2008) [30]. As the extraction and partial purification of the enzyme were performed prior to the initiation of the present study, these preparatory steps were excluded from the experimental objectives. The previously prepared tyrosinase was stored in working aliquots, and a fresh aliquot was thawed on each assay day. The previously prepared tyrosinase was subsequently used as the enzyme source for evaluating the tyrosinase inhibitory activity of the tested samples.

To measure the inhibitory activity of tyrosinase, the initial‐velocity method was used in the continuous‐spectrophotometric variant adapted to mushroom tyrosinase (EC 1.14.18.1) and L‐tyrosine, following the spectrophotometric‐methodology framework of García‐Molina et al. (2007) [31].

Briefly, partially purified mushroom tyrosinase (20 µg in 75 µL) was pre‐incubated alone (control) and with each concentration of the extracts or essential oils (15 µL in DMSO) for 5 min in 50 mM sodium phosphate buffer (pH 6.8) containing 0.1 mM SDS. The concentrations ranged from 0 to 50 µg/mL for the extracts and from 0 to 1 mg/mL for the essential oils. The reaction was initiated by adding 1.35 mL of 0.5 mM L‐tyrosine in the same buffer, and the dopachrome accumulation at 475 nm was monitored at 1 min intervals for 10 min. The initial velocity (v_0_) was taken as the slope ΔA/Δt over the linear portion of the progress curve; percent inhibition was calculated as:

$$\mathrm{PI}\;\%\;\left(v_{0},\;\mathrm{control}-v_{0},\;\mathrm{sample}\right)/\left(v_{0},\;\mathrm{control}\right)\times 100$$

Kojic acid was used as a positive reference inhibitor. Curves were accepted only if the kojic acid IC_50_ fell within a pre‐established acceptance window. IC_50_ values were derived from the linear fit of PI % versus extract concentration, experiments were performed in triplicate (*n* = 3).

### 2.10 Molecular Docking

#### 2.10.1 Selection and Preparation of Target Proteins

To elucidate the molecular interactions and binding mechanisms of the bioactive compounds identified in this investigation, the execution of an in silico molecular docking analysis, utilizing tyrosinase as the therapeutic target, was conducted, which may provide insight into the potential binding affinities. The acquisition of the three‐dimensional (3D) crystal structure of the enzyme was completed using the Research Collaboratory for Structural Bioinformatics (RCSB) Protein Data Bank (https://www.rcsb.org; accessed on 26 November 2025) under the PDB ID 2Y9X. Before docking, the protein structure was prepared and refined using BIOVIA Discovery Studio Visualizer (https://discover.3ds.com/discovery‐studio‐visualizer‐download; accessed on 26 November 2025). The preprocessing workflow included removal of crystallographic water molecules, addition of polar hydrogen atoms, reconstruction of missing residues, and assignment of Gasteiger partial charges [32]. The optimized receptor structure was then saved in PDB format and converted into PDBQT format for subsequent docking simulations.

#### 2.10.2 Ligand Preparation

The retrieval of the three‐dimensional (3D) conformations of the selected ligands was performed using the PubChem database, a procedure that may facilitate subsequent structural analysis (https://pubchem.ncbi.nlm.nih.gov; accessed on 26 November 2025) in SDF format. The molecular structures underwent cleaning, energy minimization, and conversion into their optimized three‐dimensional forms via Avogadro (Source Forge; accessed on 26 November 2025). Assignment of the appropriate protonation states was performed, and the implementation of full structural optimization was undertaken before docking, which may contribute to the predictive accuracy of the simulation [33].

#### 2.10.3 Molecular Docking Procedures

Molecular docking simulations were performed using AutoDock Vina implemented in the PyRx 0.8 virtual screening platform (accessed on 25 November 2025). AutoDock Vina employs a gradient‐based conformational search algorithm coupled with an empirical scoring function to predict the most favorable ligand–protein binding conformations and estimate their corresponding binding free energies (ΔG, kcal/mol) [34].

The prepared tyrosinase receptor (PDB ID: 2Y9X) and the optimized ligand structures were imported into PyRx. The receptor was processed using the “Make Macromolecule” module, whereas ligands were prepared with the “Make Ligand” utility to generate PDBQT files suitable for docking calculations. During receptor preparation, crystallographic water molecules and non‐essential heteroatoms were removed, while polar hydrogen atoms and Kollman charges were added using AutoDock Tools 1.5.7. Ligands were assigned Gasteiger charges, and all rotatable bonds were defined prior to docking.

The docking grid box was centered on the catalytic cavity of tyrosinase to encompass the dicopper‐containing active site and the surrounding amino acid residues involved in ligand recognition. The grid center coordinates and box dimensions were determined using BIOVIA Discovery Studio Visualizer, ensuring complete coverage of the binding pocket. The final grid parameters employed for the docking calculations are summarized in Table 1 [35].

**TABLE 1:** Grid map parameters for tyrosinase (2Y9X) used in docking.

Parameter	X	Y	Z
Grid center (Å)	−3.852	−22.283	−36.909
Grid size (Å)	24.591	18.431	20.214

To ensure adequate sampling of the conformational space, the exhaustiveness parameter was set to 8, whereas all remaining AutoDock Vina parameters were maintained at their default values. Binding affinities were expressed as predicted free binding energies (ΔG, kcal/mol), with more negative values indicating more favorable predicted ligand–protein interactions.

The reliability of the docking protocol was assessed through redocking of the co‐crystallized ligand into the active site of tyrosinase (PDB ID: 2Y9X). The docked ligand pose was subsequently superimposed onto the experimental crystallographic conformation using PyMOL Molecular Graphics System (Version 3.1, Schrödinger, LLC), and the root mean square deviation (RMSD) between the two conformations was calculated. The redocking procedure yielded an RMSD value of 0.50 Å, which is well below the generally accepted threshold of 2.0 Å, confirming the accuracy of the docking protocol and the suitability of the selected grid parameters for predicting ligand binding within the tyrosinase active site.

Following docking, the predicted ligand–protein complexes were analyzed using BIOVIA Discovery Studio Visualizer. Two‐dimensional and three‐dimensional interaction diagrams were generated to identify the principal non‐covalent interactions involved in complex stabilization, including conventional hydrogen bonds, hydrophobic contacts, π–π stacking, π–alkyl interactions, electrostatic interactions, and van der Waals contacts. These interaction profiles were subsequently used to characterize the binding modes of the investigated phytochemicals within the catalytic pocket of tyrosinase.

Because molecular docking provides only a computational prediction of ligand binding, the calculated binding energies were interpreted qualitatively and used primarily to compare the relative binding potential of the investigated compounds. Therefore, the docking results were considered complementary to the experimental biological assays and were not interpreted as direct evidence of inhibitory potency or correlated quantitatively with the IC_50_ values of the crude extracts.

Following docking, the predicted ligand–protein complexes and their spatial orientations within the catalytic pocket were visualized and analyzed using BIOVIA Discovery Studio Visualizer. Key non‐covalent interactions, including hydrogen bonds, hydrophobic contacts, π–π stacking, and electrostatic interactions with critical active‐site residues, were identified and characterized [36].

### 2.11 Statistical Analysis

All experiments were performed using three independent measurements, and the results are expressed as mean ± standard deviation (SD). Statistical analyses were performed using IBM SPSS Statistics. For DPPH, ABTS, phosphomolybdenum (PM), FRAP, and tyrosinase inhibition, the effects of sample type (essential oil versus hydroalcoholic extract), harvesting season (spring, summer, autumn, and winter), and their interaction were evaluated using two‐way analysis of variance (ANOVA). Because IC_50_ values are positive and may exhibit variance heterogeneity across concentration‐dependent biological responses, IC_50_ data were additionally examined after logarithmic transformation before factorial analysis. Homogeneity of variances was assessed using Levene's test, while residual distributions were examined using the Shapiro–Wilk test and Q–Q plots. When significant effects were detected, Tukey's honestly significant difference (HSD) test was used for multiple comparisons. For total phenolic content (TPC) and total flavonoid content (TFC), which were determined for the essential‐oil samples across the four harvesting seasons, seasonal differences were evaluated using one‐way ANOVA followed by Tukey's HSD test. Statistical significance was established at *p* < 0.05. Effect sizes for factorial ANOVA were expressed as partial eta squared (ηp^2^).

## 3 Results and Discussion

### 3.1 Climatic and Physiological Context of the Collection Site

Because the biosynthesis of phenolic and volatile secondary metabolites in Mediterranean aromatic shrubs is tightly coupled to environmental cues, the seasonal phytochemical patterns observed for *A. herba‐alba* in the present study must be interpreted in light of the climatic context of the Aflou collection site. Plant material was harvested in the Aflou zone, north‐western Laghouat wilaya (34°06′50″ N, 2°05′50″ E), at approximately 1096 m above sea level. NASA POWER reanalysis data (01/01/2023–31/12/2023) for the corresponding grid cell (34.0663°N, 2.3385°E) describe a strongly continental, semi‐arid climate with cold winters and hot, dry summers. The mean air temperature at 2 m (T2M) ranged from 5.32°C to 32.43°C, with daily maxima of 43.36°C and minima of −5.20°C, giving an annual thermal amplitude greater than 48°C. All‐sky surface shortwave downward irradiance (ALLSKY_SFC_SW_DWN) was highest in late spring (27.76 MJ·m^−^^2^·d^−^^1^ in May) and remained high throughout summer (≥26.93 MJ·m^−^^2^·d^−^^1^) before declining to 9.86 MJ·m^−^^2^·d^−^^1^ in December. Corrected precipitation was very low overall; May (1.32 mm·d^−^^1^) and September (0.89 mm·d^−^^1^) were the rainiest months, while July and August were essentially dry (0.03 and 0.10 mm·d^−^^1^, respectively). Relative humidity at 2 m (RH2M) showed the inverse pattern, dropping to 17.48% in July and rising to 65.89% in January, and surface soil wetness fell to 0.15 m^3^·m^−^^3^ in July and 0.12 m^3^·m^−^^3^ in August, confirming the severity of the summer water deficit. Mean wind speed at 2 m (WS2M) ranged from 2.82 to 4.22 m·s^−^^1^, with the highest values in May and November.

For the harvesting calendar adopted in this study, this means the *March* (spring) sample experienced moderate temperatures (T2M = 13.45°C, daily max 29.47°C), declining humidity (RH2M = 43.96%) and increasing irradiance (20.91 MJ·m^−^^2^·d^−^^1^); the *July* (summer) sample experienced the most extreme combination of heat (T2M = 32.43°C; daily max 43.36°C), low RH2M (17.48%), near‐zero precipitation (0.03 mm·d^−^^1^) and a minimum surface soil wetness (0.15 m^3^·m^−^^3^); the *September* (autumn) sample was collected under moderate–high irradiance (20.09 MJ·m^−^^2^·d^−^^1^), temperatures of 25.16°C and the autumn precipitation peak (0.89 mm·d^−^^1^); the *November* (winter) sample was exposed to cool conditions (T2M = 13.46°C, T2M_MIN = 3.44°C) and a marked drop in irradiance (13.14 MJ·m^−^^2^·d^−^^1^), with frost episodes absent yet possible at dawn. These seasonal windows of heat–drought co‐stress in summer and irradiance–temperature shifts in winter constitute the abiotic framework against which the metabolite fluctuations described below should be read.

### 3.2 Total Phenolic and Flavonoid Contents

The seasonal variation of total phenolic contents (TPC) and flavonoids (TFC) in *A. herba‐alba* hydroalcoholic extracts was investigated, and the results are illustrated in Table 2 and Figure S1.

**TABLE 2:** Seasonal variation in total phenolic and total flavonoid contents of *A. herba‐alba* samples.

Season	TPC (mg GAE/g DW)	TFC (mg RE/g DW)
Spring	86.83 ± 0.57	19.21 ± 0.07
Summer	126.72 ± 0.34	37.00 ± 0.55
Winter	110.67 ± 1.47	23.87 ± 0.66
Autumn	42.11 ± 1.16	12.22 ± 0.11

*Note*: Values are expressed as mean ± standard deviation (SD) of three independent measurements (*n* = 3). TPC is expressed as mg gallic acid equivalents (GAE) g^−^^1^ dry weight (DW), whereas TFC is expressed as mg rutin equivalents (RE) g^−^^1^ DW. Different superscript lowercase letters within the same column indicate significant differences among harvesting seasons according to Tukey's HSD post hoc test (*p* < 0.05).

TPC values ranged from 42.11 ± 1.16 to 126.72 ± 0.34 mg GAE·g^−^^1^ dw and TFC from 12.22 ± 0.11 to 37.00 ± 0.55 mg RE·g^−^^1^ dw across the four seasons. The highest phenolic and flavonoid contents were obtained in summer (TPC = 126.72 ± 0.34 mg GAE·g^−^^1^ dw; TFC = 37.00 ± 0.55 mg RE·g^−^^1^ dw), followed by winter (TPC = 110.67 ± 1.47 mg GAE·g^−^^1^ dw; TFC = 23.87 ± 0.66 mg RE·g^−^^1^ dw), spring (TPC = 86.83 ± 0.57 mg GAE·g^−^^1^ dw; TFC = 19.21 ± 0.07 mg RE·g^−^^1^ dw) and autumn (TPC = 42.11 ± 1.16 mg GAE·g^−^^1^ dw; TFC = 12.22 ± 0.11 mg RE·g^−^^1^ dw). The simultaneous summer maximum of TPC and TFC co‐occurs with the period of greatest photosynthetically active radiation (ALLSKY_SFC_SW_DWN = 26.93 MJ·m^−^^2^·d^−^^1^), highest air temperature (T2M_MAX = 43.36°C), lowest relative humidity (17.48%) and lowest surface soil wetness (0.15 m^3^·m^−^^3^) recorded in the Aflou grid cell. Seasonal variation in phenolic content has been documented across a wide range of medicinal species, where phenological stage and harvest date consistently emerge as the principal drivers of TPC and TFC fluctuations [9, 10, 11, 12]. The autumn minimum observed here (TPC = 42.11 mg GAE·g^−^^1^ dw; TFC = 12.22 mg RE·g^−^^1^ dw) is consistent with the dilution effect typically observed at the end of the active vegetative growth phase, when assimilated carbon is partly redirected to post‐flowering growth and reproductive structures rather than to secondary metabolism [9, 11, 12]. The second, sizeable winter peak (TPC = 110.67 mg GAE·g^−^^1^ dw) observed despite the substantial autumn drop further supports a phenological‐stage dependence rather than a purely thermal one, since phenological surveys of related *Artemisia* and other Asteraceae have shown that pre‐vernal stages under cool, low‐irradiance conditions retain, or even increase, phenolic loads relative to mid‐autumn [10, 11, 12].

Our TPC values are consistent with previously reported data for *A. herba‐alba* from comparable semi‐arid environments. A Tunisian accession yielded 123.95 ± 4.3 g GAE·kg^−^^1^ dw [37]; complementary Tunisian samples gave a markedly lower TPC of 27.65 ± 0.08 mg GAE·g^−^^1^ dw [38]; Moroccan populations reached 217.60 ± 0.58 mg GAE·g^−^^1^ dw [39]; and Algerian mountainous accessions gave 104.54 ± 0.35 mg GAE·g^−^^1^ dw [40]. For TFC, the Aflou range (12.22–37.00 mg RE·g^−^^1^ dw) was higher than the Algerian mountainous sample (2.06 ± 0.03 mg RE·g^−^^1^ dw) [40], lower than the Moroccan population (43.59 ± 0.06 mg QE·g^−^^1^ dw) [39] and close to the Tunisian range (13.96 ± 0.07 mg RE·g^−1^ dw) [38]. This dispersion is consistent with the reported impact of geographical origin, climatic year, and extraction solvent on TPC and TFC of *A. herba‐alba* accessions in North Africa [7, 10, 41].

### 3.3 Essential Oil Yield

**FIGURE 1:**
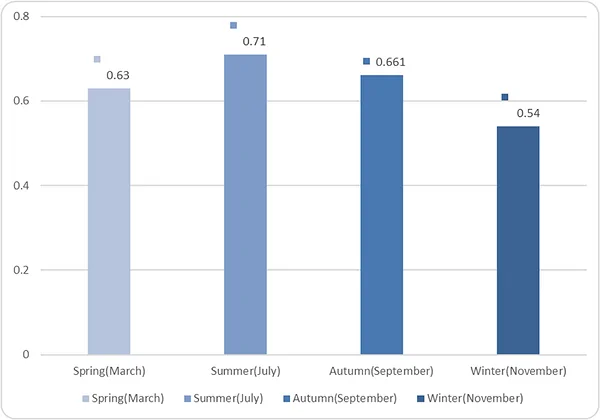
Seasonal variation in essential oil yields of *A. herba‐alba* plants.

The seasonal yield of *A. herba‐alba* essential oil in our study, as displayed in Figure 1, ranged from 0.54% to 0.71%, with intermediate values of 0.63% in March and 0.66% (w/w) in September. The summer maximum coincides with the period of minimum soil wetness and maximum irradiance, while the minimum coincides with low temperatures and reduced irradiance, consistent with the documented dependence of glandular trichome density and terpene yield on phenological stage [9, 11, 12, 42]. The Aflou yields (0.54%–0.71%) fall within the 0.2%–0.9% range previously reported for Algerian *A. herba‐alba* populations [2], and agree with single‐harvest values from Algeria of 0.6% [43], 0.7% [44], and 0.8% [45], as well as with the 0.68%–1.93% range reported from southern Tunisia [46]. Higher values (1.85%–2.35%) were observed in a Moroccan study in which seasonal yields varied between April, July, and October harvests [42], the somewhat lower values obtained here may reflect both the drier, more continental climate of the Aflou high plateau (annual precipitation pattern with mostly dry summer) and the narrower summer‐peak distribution recorded for Algerian chemotypes [2]. Taken together, these observations confirm a moderately variable, climatically sensitive but regionally consistent oil content for *A. herba‐alba* [2, 42, 46].

### 3.4 Liquid Chromatography–Mass Spectrometry Profiling of Phenolic Extracts

The results presented in Table 3 summarize the LC–MS identification and quantification of phenolic compounds in the hydroalcoholic extracts of *A. herba‐alba* collected during different seasons. The obtained chromatograms are reported in Figures S2 and S3.

**TABLE 3:** LC–MS identification and quantification of phenolic compounds in *A. herba‐alba* extracts collected during different seasons.

Compound name	RT (min)	Spring µg·g^−^^1^	Summer µg·g^−^^1^	Autumn µg·g^−^^1^	Winter µg·g^−^^1^
Gallic acid	2.91	0.20	0.17	0.22	0.26
Chlorogenic acid	2.93	0.28	0.30	0.31	0.57
Gentisic acid	3.11	3.18	3.19	3.17	3.27
Caffeic acid	3.54	1.92	1.96	1.68	1.93
Syringic acid	3.57	1.24	1.58	1.47	1.89
Vanillic acid	3.72	2.48	2.30	1.99	2.50
*p*‐Coumaric acid	4.36	0.53	0.46	0.46	0.56
Ferulic acid	4.64	1.83	1.80	2.77	1.63
Salicylic acid	7.26	3.06	—	—	3.67
Protocatechuic acid	7.91	0.61	—	—	1.00
*trans*‐Cinnamic acid	10.09	5.32	5.95	5.52	8.83
Quercetin	10.04	7.98	11.42	4.83	11.48
Kaempferol	12.19	2.65	4.26	2.71	3.03
Morin	11.92	0.96	1.13	0.72	1.19
Naringenin	11.00	139.89	126.56	104.94	145.74
Chrysin	13.71	1.29	1.31	0.55	0.91
Luteolin	14.03	16.06	19.41	8.88	15.68
Biochanin A	13.62	0.25	0.26	0.21	0.27
Vanillin	4.93	52.94	45.20	39.51	43.12

LC–MS analysis identified 19 phenolic compounds grouped into phenolic acids (11 compounds), flavonol aglycones (three), flavanone aglycones (one), flavone aglycones (two), isoflavone aglycones (one) and other phenolics (one). The phenolic acid profile (gentisic, vanillic, caffeic, syringic, *p*‐coumaric, ferulic, salicylic, protocatechuic and *trans*‐cinnamic acids, plus minor gallic and chlorogenic acids) broadly consistent with the North African *A. herba‐alba* pattern previously documented in multi‐provenance comparisons [7, 41, 47]. *Trans‐*cinnamic acid was the most abundant phenolic acid (5.32–8.83 µg·g^−^^1^) and showed its absolute winter maximum (8.83 µg·g^−^^1^) under the cool, low‐irradiance conditions observed for the November harvest, consistent with phenological studies reporting increased accumulation of phenylpropanoid precursors in late autumn in medicinal Asteraceae [11, 12]. Among flavonols, quercetin ranged from 4.83 µg·g^−^^1^ (autumn) to 11.48 µg·g^−^^1^ (winter), kaempferol from 2.65 to 4.26 µg·g^−^^1^ and morin from 0.72 to 1.19 µg·g^−^^1^. These flavonols (quercetin, luteolin, kaempferol) are compatible with the main flavonoid pattern reported by Alqubelat and Atrooz (2024) [48] and generally agree with [47], although our samples were more dominated by flavanones and vanillin than those studies. The flavanone naringenin (104.94–145.74 µg·g^−^^1^) and the “other phenolic” vanillin (39.51–52.94 µg·g^−^^1^) were by far the two most abundant individual compounds across all seasons, with naringenin peaking in winter (145.74 µg·g^−^^1^) and vanillin in spring (52.94 µg·g^−^^1^). The flavones luteolin (8.88 µg·g^−^^1^ in autumn to 19.41 µg·g^−^^1^ in summer) and chrysin (0.55–1.31 µg·g^−^^1^) showed clear summer maxima, consistent with light‐driven induction in summer under the highest all‐sky shortwave irradiance of the year (≥26.87 MJ·m^−^^2^·d^−^^1^) [11, 12]. The isoflavone biochanin A was present at very low concentrations throughout the year (0.21–0.27 µg·g^−^^1^). Seasonal variations detected for minor acids such as salicylic and protocatechuic acids (present only in spring and winter) and chlorogenic and gallic acids (maximal in winter) likely reflect local micro‐edaphic and stage‐specific signals, as previously suggested by HPLC–DAD investigations of *A. herba‐alba* harvested at three different dates [41]. Overall, our seasonal profiling showed a phenolic‐class distribution in which phenolic acids were predominant, followed by flavonols and flavones, a pattern broadly consistent with previous reports [7, 47, 48], with the notable feature of a high flavanone/vanillin load that has not been previously emphasized and may constitute a chemotypic particularity of the Aflou population under its continental high‐plateau climate.

### 3.5 Gas Chromatography–Mass Spectrometry Profiling of Essential Oils

The results presented in Tables 4 and 5 summarize the chemical composition *A. herba‐alba* essential oils analyzed by GC–MS during four different seasons (spring, summer, autumn, and winter) and the seasonal variation of major chemical classes in *A. herba‐alba* essential oils (%) (Figures S4‐S7).

**TABLE 4:** GC–MS identification and quantification of volatile compounds in *A. herba‐alba* essential oils collected during different season.

Compound name	RT^(min)^	RI^Cal^	RI ^lit^	Spring %	Summer %	Autumn %	Winter %
Santolinatriene	7.416	901	906	—	—	—	0.43
*p*‐Mentha‐3,8‐diene	7.915	914	910	0.26	—	—	—
*α*‐Thujene	8.095	919	924	—	0.76	0.29	0.93
*α*‐Pinene	8.324	925	932	1.44	—	0.29	—
Camphene	8.879	939	946	5.21	1.67	—	2.03
Sabinene	9.878	964	969	0.49	0.21	—	0.08
*β*‐Pinene	9.977	967	974	—	—	—	0.28
Yomogi alcohol	10.981	992	999	—	0.17	—	1.53
*γ*‐Terpinene	13.540	1009	1004	—	—	—	0.23
Isopulegol	11.938	1015	1010	—	—	—	0.28
*o*‐Cymene	12.034	1017	1020	—	0.78	—	0.74
*p*‐Cymene	12.037	1017	1022	1.28	—	0.38	—
1,8‐Cineole	12.350	1023	1026	**12.35**	**7.57**	—	**10.9**
*β*‐Ocimene	7.422	1026	1032	0.63	0.27	0.29	3.18
Hotrienol	12.577	1029	1042	0.3	0.27	—	2.96
Artemisia alcohol	14,676	1076	1080	—	—	—	2.15
Linalool	15.417	1092	1095	—	—	—	0.50
*β*‐Thujone	15.579	1108	1114	7.85	9.77	0.43	3.38
α‐Thujone	16.139	1096	1112	**15.61**	**10.14**	1.26	4.03
Isophorone	16.295	1111	1118	0.42	0.59	—	—
*p*‐Menth‐2‐en‐1‐ol	16.382	1113	1120	0.56	0.82	—	0.25
Chrysanthenone	16.515	1116	1124	**10.55**	**15.6**	0.44	4.2
Sabinol	17.142	1130	1127	1.32	—	—	0.54
*trans*‐Pinocarveol	17.145	1130	1135	—	2.93	—	—
Sabinyl acetate	17.240	1132	1129	0.47	—	—	—
Camphor	17.401	1135	1141	**30.19**	**28.89**	2.57	20.5
Pinocarvone	18.229	1150	1160	3.16	3.4	—	—
Myrtenal	18.230	1153	1164	—	—	—	1.34
Borneol	18.332	1155	1168	2.91	4.19	0.45	2.87
*α*‐Phellandren‐8‐ol	18.48	1160	1070	0.36	0.53	—	0.14
*trans*‐*β*‐Terpineol	18.732	1164	1172	—	—	—	3.19
4‐Terpinenol	18.945	1169	1174	0.92	1.05	—	0.97
Myrtenol	19.811	1187	1194	0.73	1.01	—	0.51
Verbenone	20.384	1200	1204	0.49	0.78	—	—
Nordavanone	21.314	1220	1227	—	—	0.62	—
*trans*‐Chrysanthenyl acetate	22.815	1246	1235	—	—	—	1.00
*cis*‐Chrysanthenyl acetate	22.815	1253	1261	0.18	0.38	—	—
Bornyl acetate	23.912	1278	1284	0.2	0.56	—	0.48
Thymol	24.231	1285	1289	—	—	—	3.54
*trans*‐Pinocarveol	24.525	1291	1294	—	0.15	—	—
Carvacrol	24.676	1294	1298	—	1.88	1.21	**21.73**
1,6‐Dimethylhepta‐1,3,5‐triene	25.038	1303	1300	—	0.61	—	—
1‐Ethyl‐5,5‐dimethylcyclopenta‐1,3‐diene	25.299	1309	1305	0.91	1.97	—	0.36
Eugenol	26.986	1348	1356	—	—	0.99	—
(Z)‐Jasmone	28.750	1388	1392	0.25	—	0.63	0.32
*cis*‐Davanafuran	29.510	1406	1414	—	—	1.67	—
Davana ether	32.686	1483	1487	—	—	4.8	0.22
Davana ether 2	33.501	1503	1491	—	—	**41.15**	0.47
Artedouglasia oxide C	33.852	1512	1522	—	—	1.26	—
Vinylbifuranone	34.871	1538	1530	—	—	0.93	—
Artedouglasia oxide A	35.297	1549	1534	—	—	0.2	—
Isovalencenyl formate	36.004	1567	1560	—	—	6.6	—
Trimethylcyclopropazulenol	36.039	1568	1566	—	1.32	—	0.74
Methylfuranheptenone	36.372	1577	1572	—	—	1.19	0.30
Guaia‐4,6‐diene	36.548	1581	1580	—	—	0.87	—
Trimethylcyclohexenylcarboxaldehyde	37.000	1593	1595	—	—	2.19	—
Dicyclohexylmethanone	37.511	1606	1600	—	—	1.49	—
Caryophyllene oxide	37.871	1616	1610	—	—	3.85	—
*trans*‐Longipinocarveol	38.282	1627	1622	—	—	1.47	—
*γ*‐Muurolene	38.408	1630	1633	—	—	2.43	—
*β*‐Eudesmol	38.743	1639	1650	—	—	1.68	—
*β*‐Guaiene	38.909	1644	1648	—	—	3.09	—
2‐hydroxydiphenyl methane	39.591	1662	1658	—	—	2.98	—
Steviol/hydroxydehydrostevic acid	39.724	1666	1660	—	—	**6.07**	—
*β*‐Copaen‐4α‐ol	40.063	1675	1674	—	—	0.5	—

Abbreviations: RI^Cal^.: Calculated retention indices; RI^Lit^.: Literature retention indices; RT^(min):^ retention time ‐: Not detected.

**TABLE 5:** Seasonal variation of major chemical classes in *A. herba‐alba* essential oils (%).

Chemical class	Spring	Summer	Autumn	Winter
Monoterpene hydrocarbons	9.31%	3.69%	1.25%	7.90%
Oxygenated monoterpenes	88.21%	90.0%	11.83%	86.65%
Sesquiterpene hydrocarbons	—	—	6.39%	—
Oxygenated sesquiterpenes	—	1.32%	55.64%	1.43%
Others (artefacts, phenylpropanoids, diterpenes, unidentified & non‐terpene compounds)	1.52%	3.11%	19.16%	0.82%
**Total identified**	**99.04%**	**98.12%**	**94.27%**	**96.80%**

GC–MS analysis of the four seasonal oils detected 65 compounds, with 29 in spring, 33 in summer, 31 in autumn, and 42 in winter, in line with the chemical complexity reported in earlier multi‐season surveys of *A. herba‐alba* [1, 42]. The distribution of the main chemical classes varied markedly among the seasons. Oxygenated monoterpenes predominated in spring (88.21%), summer (90.0%), and winter (86.65%), but decreased sharply in autumn (11.83%). Oxygenated sesquiterpenes were not detected in spring, accounted for 1.32% in summer, increased substantially to 55.64% in autumn, and declined to 1.43% in winter. Monoterpene hydrocarbons represented 9.31% in spring, 3.69% in summer, 1.25% in autumn, and 7.90% in winter, whereas sesquiterpene hydrocarbons were detected only in autumn (6.39%). Other compounds, including artefacts, phenylpropanoids, diterpenes, unidentified compounds, and non‐terpene compounds, accounted for 1.52% in spring, 3.11% in summer, 19.16% in autumn, and 0.82% in winter.

The spring oil, dominated by camphor (30.19%), *α*‐ and *β*‐thujone (23.46% combined), 1,8‐cineole (12.35%) and chrysanthenone (10.55%), reproduced the classical Algerian “camphor–thujone” chemotype described by Vermin et al. (1995), Dob and Benabdelkader (2006) [44, 49] and is broadly congruent with the variability reported by Belhattab et al. (2014) [2] for other Algerian populations. The summer profile was similar but shifted, with camphor (28.89%), thujones (19.91%), chrysanthenone (15.6%) and the first appearance of carvacrol (1.88%) as a trace phenolic monoterpene. The Tunisian semi‐arid chemotype described by Kadri et al. (2011) [1], in which camphor, *α*‐thujone, and chrysanthenone are co‐dominant, is the closest published analogue to our summer oil. The 1,8‐cineole fraction (7.57%–12.35%), stable across all four seasons, agrees with the Jordanian populations profiled by Alqubelat and Atrooz (2024) [48] and with the cineole–camphor chemotypes of French and Palestinian origins reviewed by Kadri et al. (2011) [1], and Belhattab et al. (2014) [2], supporting the view that 1,8‐cineole synthesis is under relatively conserved genetic control in the species.

The autumn oil was qualitatively very distinctive: davana ether 2 reached 41.15%, accompanied by steviol/hydroxydehydrostevic acid (6.07%), isovalencenyl formate (6.60%), caryophyllene oxide (3.85%), *β*‐guaiene (3.09%), 2‐hydroxydiphenyl methane (2.98%), artedouglasia oxide A and C, *β*‐eudesmol, and several other minor oxygenated sesquiterpenes. The classical oxygenated monoterpenes almost disappeared (camphor 2.57%, *α*‐thujone 1.26%, *β*‐thujone 0.43%). This transient “autumn sesquiterpene/davana chemotype” has not, to our knowledge, been reported for *A. herba‐alba* from other North African stations, although reports of marked seasonal shifts toward sesquiterpenes exist for other aromatic Asteraceae under post‐flowering, post‐stress conditions [11, 12, 42]. Accordingly, the autumn chemical class table exhibits a clear reversal of the spring/summer oxygenated‐monoterpene dominance, indicating a phenological switch of terpene biosynthesis between the active summer period and the post‐monsoonal recovery period, as illustrated by the September precipitation peak (0.89 mm·d^−1^) and the rebounding soil wetness (GWETTOP increasing from 0.12 to 0.28 m^3^·m^−3^) [11, 12, 42].

The winter oil recovered a classical oxygenated‐monoterpene distribution (86.79%), but with a pronounced rise of phenolic monoterpenes: carvacrol reached 21.73%, thymol 3.54%, and 1,8‐cineole 10.9%, while camphor fell to 20.5% and *α*‐/*β*‐thujone to 7.41% combined. The appearance of a phenolic‐monoterpene‐rich winter chemotype conforms with the phytochemical plasticity observed in other *Artemisia* accessions, where camphor, thujone and 1,8‐cineole co‐occur with phenolic terpenes [19, 26, 50, 51]. Although a carvacrol‐rich chemotype has rarely been reported for Algerian *A. herba‐alba*, the winter climatic window of the Aflou site (cool nights with T2M_MIN approaching 0.68°C in December and low winter soil wetness) is precisely the type of low‐temperature, low‐irradiance regime that has been associated experimentally with a shift from thujone/camphor to phenolic‐monoterpene biosynthesis in related taxa [11, 19, 42]. These observations reinforce both the regional consistency of camphor–thujone chemotypes in Algeria [2, 3, 4, 5, 44, 45, 49] and the seasonal plasticity of the species [1, 42, 46].

### 3.6 Seasonal Variation in Antioxidant Activity

The antioxidant activity of *A. herba‐alba* essential oil and ethanolic extract from the Laghouat region were determined seasonally, and the results are summarized in Tables 6 and 7 and Figures S8‐S11.

**TABLE 6:** Antioxidant activity of *A. herba‐alba* essential oils and hydroalcoholic extracts according to harvesting season.

Sample	Season	DPPH IC_50_ (µg/mL)	ABTS IC_50_ (µg/mL)	PM (mmol/g)	FRAP (mmol/g)
Essential oil	Spring	122.08 ± 1.46	111.14 ± 1.52	0.014 ± 0.000	0.094 ± 0.001
Hydroalcoholic extract	Spring	52.70 ± 0.80	48.32 ± 0.25	0.147 ± 0.029	0.214 ± 0.016
Essential oil	Summer	159.21 ± 1.49	155.01 ± 5.29	0.042 ± 0.000	0.012 ± 0.000
Hydroalcoholic extract	Summer	29.40 ± 0.10	27.16 ± 0.07	0.331 ± 0.024	0.436 ± 0.001
Essential oil	Winter	67.79 ± 0.14	69.11 ± 0.52	0.140 ± 0.007	0.187 ± 0.004
Hydroalcoholic extract	Winter	31.11 ± 0.07	28.36 ± 0.14	0.140 ± 0.073	0.287 ± 0.000
Essential oil	Autumn	126.00 ± 2.08	140.11 ± 3.05	0.033 ± 0.000	0.041 ± 0.000
Hydroalcoholic extract	Autumn	61.12 ± 1.10	63.98 ± 1.11	0.127 ± 0.012	0.122 ± 0.000

*Note*: Values are expressed as mean ± standard deviation (SD) of three independent measurements (*n* = 3). DPPH and ABTS results are expressed as IC_50_ values (µg mL^−^^1^), with lower IC_50_ values indicating stronger radical‐scavenging activity. PM and FRAP values are expressed as mmol g^−^^1^, with higher values indicating greater reducing/antioxidant capacity. Different superscript lowercase letters within the same column indicate significant differences according to Tukey's HSD post hoc test (*p* < 0.05).

**TABLE 7:** Two‐way analysis of variance (ANOVA) of the effects of sample type, harvesting season, and their interaction on antioxidant activity.

Assay	Effect	df	*F*	*p*‐value	Partial η^2^
DPPH IC_50_	Sample	1	25,670.302	<0.001	0.999
DPPH IC_50_	Season	3	2,075.019	<0.001	0.997
DPPH IC_50_	Sample × Season	3	1,743.059	<0.001	0.997
ABTS IC_50_	Sample	1	6,899.840	<0.001	0.998
ABTS IC_50_	Season	3	616.975	<0.001	0.991
ABTS IC_50_	Sample × Season	3	398.954	<0.001	0.987
PM	Sample	1	115.113	<0.001	0.878
PM	Season	3	18.378	<0.001	0.775
PM	Sample × Season	3	25.065	<0.001	0.825
FRAP	Sample	1	5,755.018	<0.001	0.997
FRAP	Season	3	900.741	<0.001	0.994
FRAP	Sample × Season	3	1,158.113	<0.001	0.995

*Note*: Sample type included essential oil and hydroalcoholic extract, while harvesting season included spring, summer, autumn, and winter. Data were analyzed using two‐way ANOVA. Results are presented as *F*‐statistics, degrees of freedom (df), *p*‐values, and partial eta squared (ηp^2^) as a measure of effect size. The sample × season interaction indicates whether the effect of harvesting season differed according to sample type.

The antioxidant activity of *A. herba‐alba* was markedly influenced by both the nature of the tested fraction and the harvesting season. The factorial analysis revealed significant effects of sample type, season, and sample × season interaction for all antioxidant parameters investigated (*p* < 0.001), demonstrating that seasonal variation was strongly dependent on whether the plant material was evaluated as an essential oil or as a hydroalcoholic extract.

For DPPH radical‐scavenging activity, the hydroalcoholic extracts exhibited substantially lower IC_50_ values than the corresponding essential oils across all seasons, indicating a markedly greater radical‐scavenging capacity. The summer extract showed the lowest numerical IC_50_ value (29.40 ± 0.10 µg mL^−^^1^), closely followed by the winter extract (31.11 ± 0.07 µg mL^−^^1^). These two extracts were not significantly different according to Tukey's HSD test (*p* = 0.616). The spring and autumn extracts showed higher IC_50_ values of 52.70 ± 0.80 and 61.12 ± 1.10 µg mL^−^^1^, respectively, and differed significantly from the summer and winter extracts. Among the essential oils, the winter sample exhibited the strongest DPPH activity (67.79 ± 0.13 µg mL^−^^1^), whereas the summer essential oil displayed the weakest activity (159.21 ± 1.49 µg mL^−^^1^). The two‐way ANOVA showed highly significant effects of sample type (*p* < 0.001), season (*p* < 0.001), and their interaction (*p* < 0.001).

A similar pattern was observed with the ABTS assay. The summer and winter extracts exhibited the lowest IC_50_ values, 27.16 ± 0.07 and 28.36 ± 0.14 µg mL^−^^1^, respectively, and were statistically indistinguishable (*p* = 0.997). The autumn extract showed a significantly higher IC_50_ (63.98 ± 1.11 µg mL^−^^1^). Among the essential oils, the winter sample showed the lowest IC_50_ (69.11 ± 0.52 µg mL^−^^1^), while the summer sample exhibited the highest value (155.01 ± 5.28 µg mL^−^^1^). The winter essential oil and autumn essential oil did not differ significantly (*p* = 0.171), whereas the remaining seasonal comparisons were significant. The significant sample × season interaction (*p* < 0.001) confirms that seasonal changes in ABTS‐scavenging capacity differed according to sample type.

The complementary PM and FRAP assays further supported the higher antioxidant potential of the hydroalcoholic extract. The highest PM value was recorded for the summer extract (0.331 ± 0.024 mmol g^−^^1^), whereas the essential oils exhibited considerably lower values. Tukey's analysis indicated that the summer extract differed significantly from all other sample‐season combinations. In contrast, several winter and non‐summer groups showed statistically comparable PM values, highlighting the importance of considering the interaction rather than season alone.

FRAP provided an even clearer separation among the tested samples. The summer extract showed the highest reducing capacity (0.436 ± 0.001 mmol g^−^^1^), followed by the winter extract (0.287 ± 0.000 mmol g^−^^1^), spring extract (0.214 ± 0.016 mmol g^−^^1^), and autumn extract (0.122 ± 0.000 mmol g^−^^1^). All eight sample × season combinations were significantly different according to Tukey's HSD test (*p* < 0.05). The summer essential oil showed the lowest FRAP value (0.012 ± 0.000 mmol g^−^^1^).

Overall, the antioxidant assays consistently indicated that the hydroalcoholic extract was more active than the essential oil. The summer extract was particularly promising, showing the lowest DPPH and ABTS IC_50_ values and the highest PM and FRAP responses. However, the seasonal response of the essential oil differed from that of the extract, with winter generally providing the most favorable antioxidant performance. This significant sample × season interaction suggests that the optimal harvesting period depends on the phytochemical fraction and the biological endpoint considered.

The seasonal changes in antioxidant activity were accompanied by substantial variation in total phenolic and flavonoid contents. The highest TPC and TFC values were recorded during summer, supporting the possibility that the enrichment of phenolic constituents contributes to the enhanced antioxidant performance of the summer extract. Nevertheless, the observed associations should not be interpreted as evidence of direct causality because antioxidant activity in a complex plant matrix may result from additive or synergistic effects among multiple constituents.

In the four antioxidant assays, extracts consistently outperformed essential oils, with the summer extract exhibiting the most potent activity (DPPH IC_50_ = 29.40 ± 0.10 µg·mL^−^^1^; ABTS IC_50_ = 27.16 ± 0.07 µg·mL^−^^1^; FRAP = 0.436 ± 0.001 mmol·g^−^^1^; PM = 0.331 ± 0.024 mmol·g^−^^1^). This extract ranking is in complete agreement with the summer TPC and TFC maxima reported above and with the well‐established phenolic‐content‐dependent reducing power of plant extracts [6, 8, 27, 52]. Our summer‐extract DPPH and ABTS values are consistent with those previously reported for *A. herba‐alba* from Tunisia (DPPH IC_50_ = 20.64 ± 0.84 µg·mL^−^^1^; ABTS = 36.60 ± 1.03 µg·mL^−^^1^) [37] and from Morocco (ethanolic extract DPPH = 27.26 ± 2.62 µg·mL^−^^1^; aqueous = 33.21 ± 1.12 µg·mL^−^^1^) [39]. Although the winter extract had lower TPC than the summer extract, it showed high activity (DPPH = 31.11 ± 0.07 µg·mL^−^^1^; ABTS = 28.36 ± 0.14 µg·mL^−^^1^), which was consistent with the higher winter load of naringenin and *trans*‐cinnamic acid, two efficient radical‐scavenging compounds [6, 8, 52].

Among essential oils, the winter oil was the most active (DPPH IC_50_ = 67.79 ± 0.14 µg·mL^−^^1^; ABTS IC_50_ = 67.79 ± 0.52 µg·mL^−^^1^; FRAP = 0.187 ± 0.004 mmol·g^−^^1^), while the summer oil was the weakest (DPPH IC_50_ = 159.21 ± 1.49 µg·mL^−^^1^; FRAP = 0.012 ± 0.000 mmol·g^−^^1^). This inversion is consistent with the seasonal chemotype shift documented above: the phenolic monoterpenes carvacrol and thymol, dominant in the winter oil, are reported to provide potent lipid‐soluble antioxidant activity in essential‐oil surveys of *Artemisia* and related genera [19, 26, 50, 51], while the summer oil dominated by camphor and thujone shows a relatively weaker radical‐scavenging capacity [1, 8, 53]. The strong winter‐oil activity observed here also exceeds values previously reported for other Algerian *A. herba‐alba* oils (DPPH IC_50_ of 7.31 ± 0.088 mg·mL^−^^1^ [1] and 1.088 mg·mL^−^^1^ [53]) but agrees well with the Moroccan value of 54.27 ± 2.04 µg·mL^−^^1^ [39], indicating that the phenolic‐rich winter chemotype of the Aflou population is among the most antioxidant‐active oils documented for this species in Algeria.

The combined pattern, summer extract > winter extract > winter oil > spring oil > summer oil > autumn oil, suggests a coherent climate‐driven biochemical rationale. The phenolic‐rich extract fraction peaks in summer, when the heat–drought–irradiance complex is most pronounced. The volatile fraction peaks in winter, when phenolic‐monoterpene biosynthesis is favored. This season‐tailored activity profile is consistent with previous multi‐season investigations of *A. herba‐alba* and related *Artemisia* taxa, where summer extracts provided the highest phenolic‐dependent radical scavenging and the volatile fractions showed winter‐maximized activity in phenolic‐monoterpene‐rich chemotypes [8, 11, 12, 42].

### 3.7 Tyrosinase Inhibitory Activity

The seasonal assessment of tyrosinase inhibitory activity within *A. herba‐alba* essential oil and ethanolic extract from the Laghouat region is summarized in Tables 8 and 9 and Figures S12 and S13. The inhibitory activity against tyrosinase was evaluated independently from the antioxidant assays using IC_50_ values as the response parameter. A clear difference was observed between the hydroalcoholic extracts and essential oils, with the extracts showing substantially lower IC_50_ values and therefore stronger tyrosinase inhibition.

**TABLE 8:** Tyrosinase inhibitory activity of *A. herba‐alba* essential oils and hydroalcoholic extracts according to harvesting season.

Sample	Season	Tyrosinase IC_50_ (µg/mL)
Essential oil	Spring	336.42 ± 4.65
Hydroalcoholic extract	Spring	3.38 ± 0.09
Essential oil	Summer	236.50 ± 3.56
Hydroalcoholic extract	Summer	2.89 ± 0.07
Essential oil	Winter	45.70 ± 1.72
Hydroalcoholic extract	Winter	3.06 ± 0.11
Essential oil	Autumn	211.45 ± 3.18
Hydroalcoholic extract	Autumn	4.63 ± 0.15

*Note*: Values are expressed as mean ± standard deviation (SD) of three independent measurements (*n* = 3). Tyrosinase inhibitory activity is expressed as IC_50_ values (µg mL^−^^1^), with lower IC_50_ values indicating stronger tyrosinase inhibition. Different superscript lowercase letters within the same column indicate significant differences according to Tukey's HSD post hoc test (*p* < 0.05). Statistical comparisons were performed among the eight sample × season combinations.

**TABLE 9:** Two‐way analysis of variance (ANOVA) of the effects of sample type, harvesting season, and their interaction on tyrosinase inhibitory activity.

Assay	Effect	df	*F*	*p*‐value	Partial η^2^
Tyrosinase IC_50_	Sample	1	42 076.628	<0.001	1.000
Tyrosinase IC_50_	Season	3	3,684.609	<0.001	0.999
Tyrosinase IC_50_	Sample × Season	3	3,670.291	<0.001	0.999

*Note*: Sample type included essential oil and hydroalcoholic extract, while harvesting season included spring, summer, autumn, and winter. Data were analyzed using two‐way ANOVA. Results are presented as *F*‐statistics, degrees of freedom (df), *p*‐values, and partial eta squared (ηp^2^) as a measure of effect size. The sample × season interaction indicates whether the effect of harvesting season differed according to sample type.

Among the hydroalcoholic extracts, the lowest numerical IC_50_ was observed for the summer sample (2.89 ± 0.07 µg mL^−^^1^), followed by the winter (3.06 ± 0.11 µg mL^−^^1^), spring (3.38 ± 0.09 µg mL^−^^1^), and autumn extracts (4.63 ± 0.15 µg mL^−^^1^). Although the summer extract presented the lowest numerical IC_50_, the differences among the seasonal extract samples were not statistically significant according to the multiple‐comparison analysis. Thus, the results indicate a consistently strong anti‐tyrosinase potential of the hydroalcoholic fraction rather than a statistically demonstrated superiority of one particular season.

In contrast, the essential oils exhibited a pronounced seasonal variation. The winter essential oil showed the lowest IC_50_ (45.70 ± 1.72 µg mL^−^^1^), indicating the strongest tyrosinase inhibition among the essential oils. This was followed by the autumn (211.45 ± 3.18 µg mL^−^^1^), summer (236.50 ± 3.56 µg mL^−^^1^), and spring essential oils (336.42 ± 4.65 µg mL^−^^1^). The seasonal differences among the essential‐oil samples were statistically significant.

Two‐way ANOVA revealed highly significant effects of sample type, harvesting season, and sample × season interaction (all *p* < 0.001). The significant interaction demonstrates that the seasonal pattern of tyrosinase inhibition differed according to the nature of the tested fraction. The hydroalcoholic extracts maintained a consistently strong inhibitory activity across seasons, whereas the essential oils displayed a pronounced improvement during winter.

The stronger anti‐tyrosinase activity of the hydroalcoholic extracts may be associated with their enrichment in polar phenolic and flavonoid constituents. The highest numerical inhibitory activity of the summer extract coincided with the highest TPC and TFC values, suggesting a possible contribution of these phytochemical groups to tyrosinase inhibition. However, this association should not be interpreted as direct evidence of causality because the extracts contain multiple constituents.

The particularly strong activity of the winter essential oil suggests that seasonal changes in its volatile composition may contribute to tyrosinase inhibition. Nevertheless, the activity of a complex essential oil cannot be attributed to individual constituents without additional mechanistic studies. Enzyme‐kinetic characterization and experiments with purified compounds would therefore be required to establish the molecular basis of the observed inhibition.

Overall, the hydroalcoholic extracts exhibited substantially stronger anti‐tyrosinase activity than the essential oils under the present experimental conditions. The significant sample × season interaction further indicates that harvesting season should be considered together with extraction type when optimizing the biological potential of the investigated plant material.

A marked seasonal and fraction‐dependent pattern may also be observed regarding the inhibition of tyrosinase. Among the essential oils, the winter variant appears to demonstrate the strongest inhibition (IC_50_ = 45.70 ± 1.72 µg·mL^−^^1^), approximately 4‐ to 7‐fold more potent than the same oil in spring (336.42 ± 4.65 µg·mL^−^^1^), summer (236.50 ± 3.65 µg·mL^−^^1^) and autumn (211.45 ± 3.18 µg·mL^−^^1^). The superior activity of the winter variant appears consistent with its elevated carvacrol content (21.73%), since phenolic monoterpenes have been shown to inhibit mushroom tyrosinase more efficiently than non‐phenolic monoterpenes such as camphor and thujone [19, 25, 26, 50, 51]. The summer, spring, and autumn oils, dominated by camphor and thujone isomers, may exhibit moderate‐to‐weak inhibition; this appears broadly consistent with the moderate anti‐tyrosinase profile reported by Cheraif et al. (2020) [54] for *A. herba‐alba* and other Algerian plants, as well as with the inhibition reported for *A. diffusa* and *A. turanica* essential oils, which, nevertheless, remained weaker than kojic acid [50, 51].

The hydroalcoholic extracts demonstrated activity at substantially lower concentrations, with IC_50_ values between 2.89 ± 0.07 µg·mL^−^^1^ (summer) and 4.63 ± 0.15 µg·mL^−^^1^ (autumn). On the in‐house partially purified tyrosinase preparation used in the present study, the summer extract evinced a potency comparable to that of kojic acid (IC_50_ = 2.59 ± 0.06 µg·mL^−^^1^) run on the same plates, supporting the within‐assay reliability of this benchmark; however, absolute numerical comparison with literature IC_50_ values obtained on commercial mushroom tyrosinase preparations should be interpreted with caution, because enzyme source, substrate concentration and buffer conditions all contribute to between‐study variability. Phenolic‐rich extracts of *A. campestris* subsp. maritima were reported by Pereira et al. (2018) [20] to reach comparable activity only at markedly higher concentrations (4.13 mg·mL^−^^1^), although this comparison is again limited by differences in the enzyme and assay system used. This observation appears consistent with the prevailing perspective, which posits that polyphenols and flavonoids constitute the primary plant‐derived tyrosinase inhibitors; their mechanism of action purportedly encompasses various processes, including the chelation of the dicopper catalytic center and the binding to pivotal residues within the active‐site pocket [13, 14, 15, 21, 22, 23]. The autumn extract evinced the weakest inhibition (IC_50_ = 4.63 ± 0.15 µg·mL^−^^1^), an observation consonant with its lowest TPC and reduced concentrations of naringenin, luteolin, and vanillin. Recent surveys have underscored the relatively infrequent activity of *A. herba‐alba* essential oils in the inhibition of tyrosinase, with only a limited subset among the 71 oils examined demonstrating significant efficacy [24]. Nevertheless, the Aflou *A. herba‐alba* extracts appear to constitute a phenolic‐rich, flavonoid‐rich subclass capable of attaining kojic‐acid‐like activity under the same within‐assay conditions, and may consequently represent promising candidates for cosmetic and dermatological applications [14, 18, 23].

### 3.8 In Silico Analysis

#### 3.8.1 Selection Rationale for the Docked Ligand Set

The ten ligands evaluated by molecular docking were intentionally selected to span the chemical space of *A. herba‐alba* seasonal oils and phenolic extracts reported in the present study and to provide a mechanistically balanced view of tyrosinase inhibition. Five compounds (camphor, *α*‐thujone, *β*‐thujone, 1,8‐cineole, carvacrol) were chosen as representative markers of the volatile fraction: camphor and the thujone isomers dominate the spring and summer chemotypes (28.89%–30.19% camphor; 19.91%–23.46% combined *α*‐/*β*‐thujone), 1,8‐cineole is conserved across all four seasons (7.57%–12.35%), and carvacrol is the principal phenolic monoterpene of the winter oil (21.73%), which also exhibited the strongest tyrosinase inhibition among essential oils (IC_50_ = 45.70 ± 1.72 µg·mL^−^^1^). Four phenolic compounds (vanillin, naringenin, luteolin, quercetin) were selected as representative markers of the hydroalcoholic extract, encompassing the dominant flavanone (naringenin, 104.94–145.74 µg·g^−^^1^), the most abundant “other phenolic” (vanillin, 39.51–52.94 µg·g^−^^1^), the principal summer‐maximizing flavone (luteolin), and a representative flavonol with documented oxidative‐enzyme interactions. Kojic acid was included as a reference inhibitor to benchmark the docking protocol, as it is the same compound used as the positive control in the in vitro tyrosinase assay. The inclusion of both fractions phenolic and volatile allows the docking output to be cross‐referenced with the season.

#### 3.8.2 Virtual Screening With PyRx

To investigate the potential interaction of the identified phytochemicals with the catalytic site of tyrosinase (PDB ID: 2Y9X), molecular docking simulations were carried out using AutoDock Vina implemented in the PyRx platform. The predicted binding free energies (ΔG, kcal·mol^−^^1^) of the investigated compounds are presented in Table 10.

**TABLE 10:** Docking scores of selected phytochemicals against tyrosinase (2Y9X).

No	Compound	Molecular weight (g/mol)	CID	Binding affinity PyRx (ΔG, kcal/mol)
1	Quercetin	302.23	5280343	−7.4
2	Luteolin	286.24	5280445	−7.3
3	Naringenin	272.25	439246	−7.2
4	Vanillin	152.15	1183	−5.8
5	Kojic acid	142.11	3840	−5.7
*6*	*α*‐Thujone	152.23	261491	−5.1
7	Carvacrol	150.22	10364	−4.9
*8*	*β*‐Thujone	152.23	91456	−4.7
9	1,8‐Cineole (Eucalyptol)	154.25	2758	−4.6
10	Camphor	152.23	2537	−4.5
11	0TR	122.12	10789	−4.5
	Tyrosinase (target enzyme)			Target enzyme (PDB ID: 2Y9X)

The calculated docking scores ranged from **−7.4 to −4.5 kcal·mol^−^^1^**, indicating variable predicted affinities of the investigated compounds toward the catalytic pocket of tyrosinase. Among the tested phytochemicals, **quercetin** exhibited the most favorable predicted binding affinity (−7.4 kcal·mol^−^^1^), followed by **luteolin** (−7.3 kcal·mol^−^^1^) and **naringenin** (−7.2 kcal·mol^−^^1^). The positive control, **kojic acid**, showed a docking score of **−5.7 kcal·mol^−^^1^**, whereas **vanillin** displayed a comparable predicted affinity (−5.8 kcal·mol^−^^1^). The remaining volatile constituents, including *α*‐thujone, carvacrol, *β*‐thujone, 1,8‐cineole, and camphor, exhibited less favorable docking scores ranging from **−5.1 to −4.5 kcal·mol^−^^1^**. The **native co‐crystallized ligand (0TR)** was redocked as part of the docking protocol validation rather than being considered a phytochemical constituent. Its predicted binding affinity (−4.5 kcal·mol^−^^1^) was used exclusively to assess the reproducibility of the docking protocol and should not be interpreted as a reference for ranking the investigated compounds.

Detailed interaction analyses of the selected complexes are presented in Table 11 and Figure 2. Quercetin, luteolin, and naringenin exhibited the most favorable predicted binding modes, establishing multiple hydrogen‐bonding and hydrophobic interactions with residues surrounding the catalytic cavity. Quercetin interacted with **GLU173, HIS178, LYS180, HIS182, and GLN44**, while luteolin and naringenin shared similar interaction networks involving **GLN44, ASN174, LYS180, and HIS178**.

**TABLE 11:** Binding affinities (ΔG) and interaction profiles of selected phytochemicals docked with Tyrosinase.

IUPAC name	ΔG (kcal·mol^−^^1^)	Interaction types observed	Key interacting residues
Quercetin	−7.4	Conventional hydrogen bond, π–alkyl, π–donor hydrogen bond	GLU173, HIS178, LYS180, HIS182, GLN44
Luteolin	−7.3	Conventional hydrogen bond, π–alkyl	GLN44, LYS180, ASN174
Naringenin	−7.2	Conventional hydrogen bond, π–alkyl, unfavorable donor–donor	LYS180, ASN174, HIS178
Vanillin (4‐hydroxy‐3‐methoxybenzaldehyde)	−5.8	π–π stacking interaction, π–alkyl interaction	PHE264, ALA286, HIS263, VAL283
Kojic acid (5‐hydroxy‐2‐(hydroxymethyl)‐4H‐pyran‐4‐one)	−5.7	π–alkyl interaction, π–π stacking interaction, conventional hydrogen bond interaction	ALA286, HIS263, VAL283
*α*‐Thujone ((1S,4R)‐4‐methyl‐1‐(propan‐2‐yl)bicyclo[3.1.0]hexan‐3‐one)	−5.1	π‐alkyl interaction, alkyl conventional hydrogen bond interaction, π‐sigma interaction	PHE264, HIS61, VAL283, HIS263, HIS85
Carvacrol (5‐isopropyl‐2‐methylphenol)	−4.9	π‐alkyl interaction, conventional hydrogen bond interaction	LYS180, GLN44
*β*‐Thujone ((1S,4R)‐4‐methyl‐1‐(propan‐2‐yl)bicyclo[3.1.0]hexan‐3‐one)	−4.7	π‐alkyl interaction, alkyl conventional hydrogen bond interaction	LYS180, HIS182
*0TR (2‐HYDROXYCYCLOHEPTA‐2,4,6‐TRIEN‐1‐ONE)*	−4.5	Conventional hydrogen bond interaction, π‐sigma	TYR201, PRO270, LEU265, PHE264

FIGURE 22D and 3D binding conformations of (A) Quercetin, (B) Luteolin, (C) Naringenin, (D) Vanillin, (E) kojic acid, (F) *α*‐Thujone, (I) Carvacrol, (G) *β*‐Thujone and (K) 0TR within the active site of tyrosinase (obtained from PyRx docking results).

**Figure:**
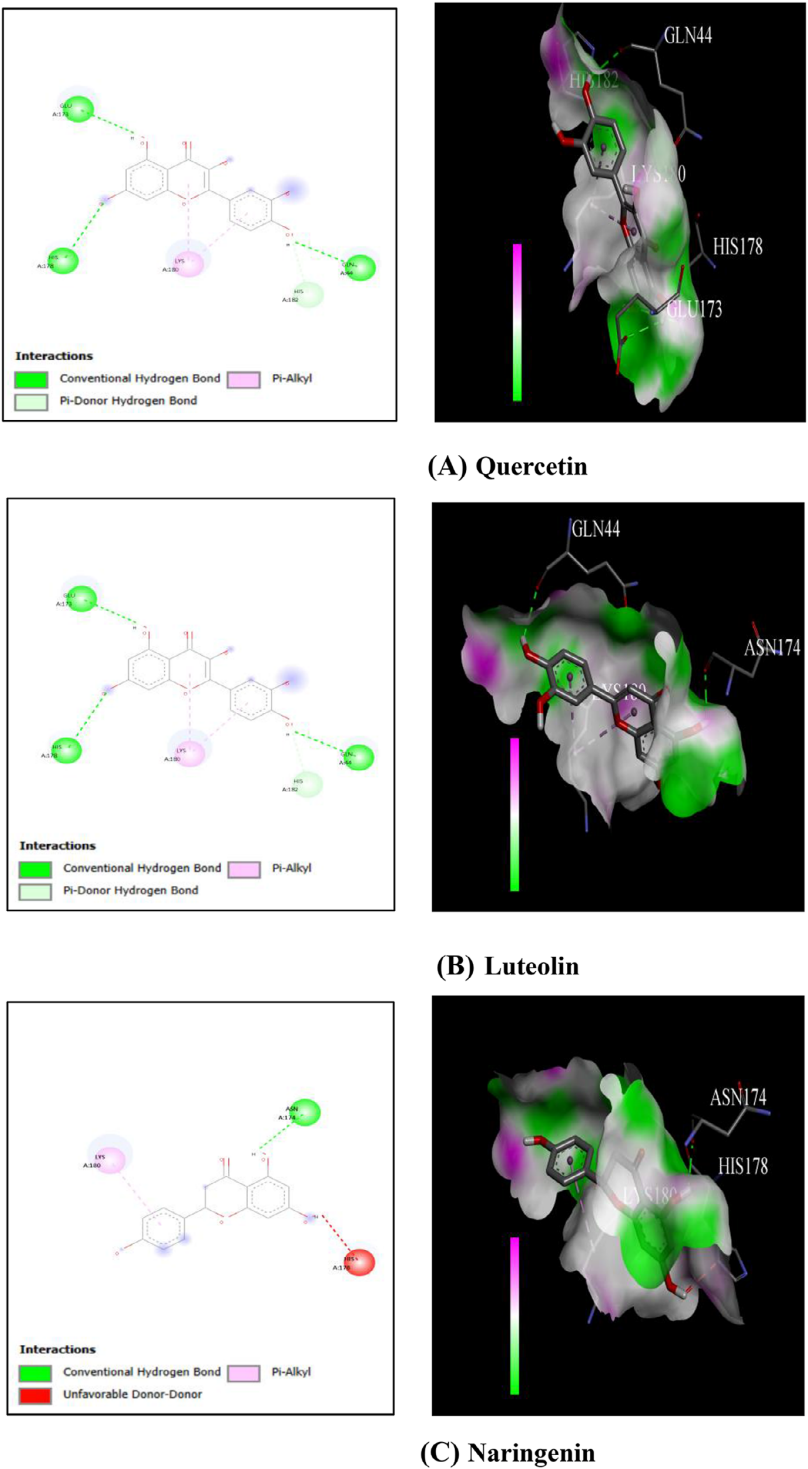

**Figure:**
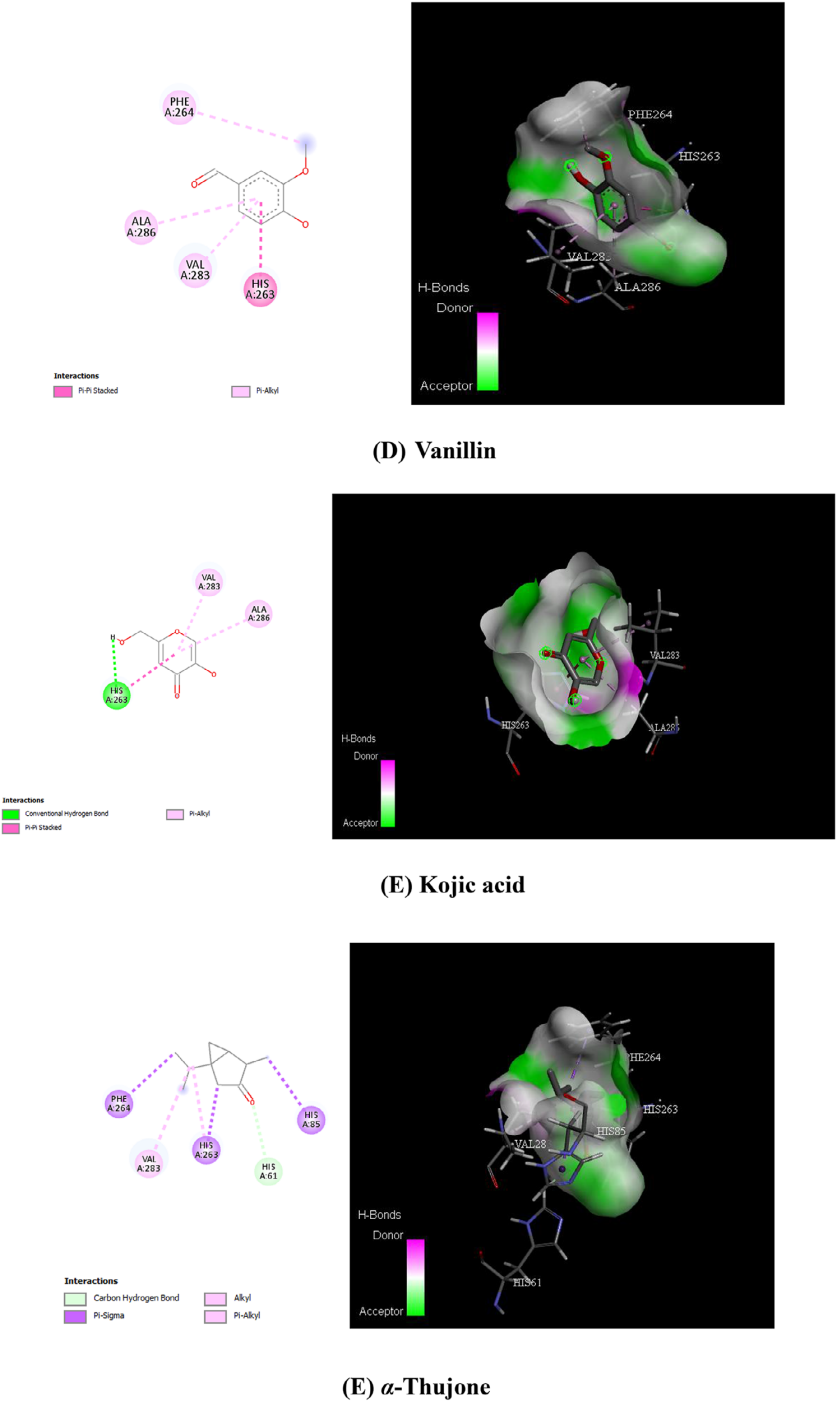

**Figure:**
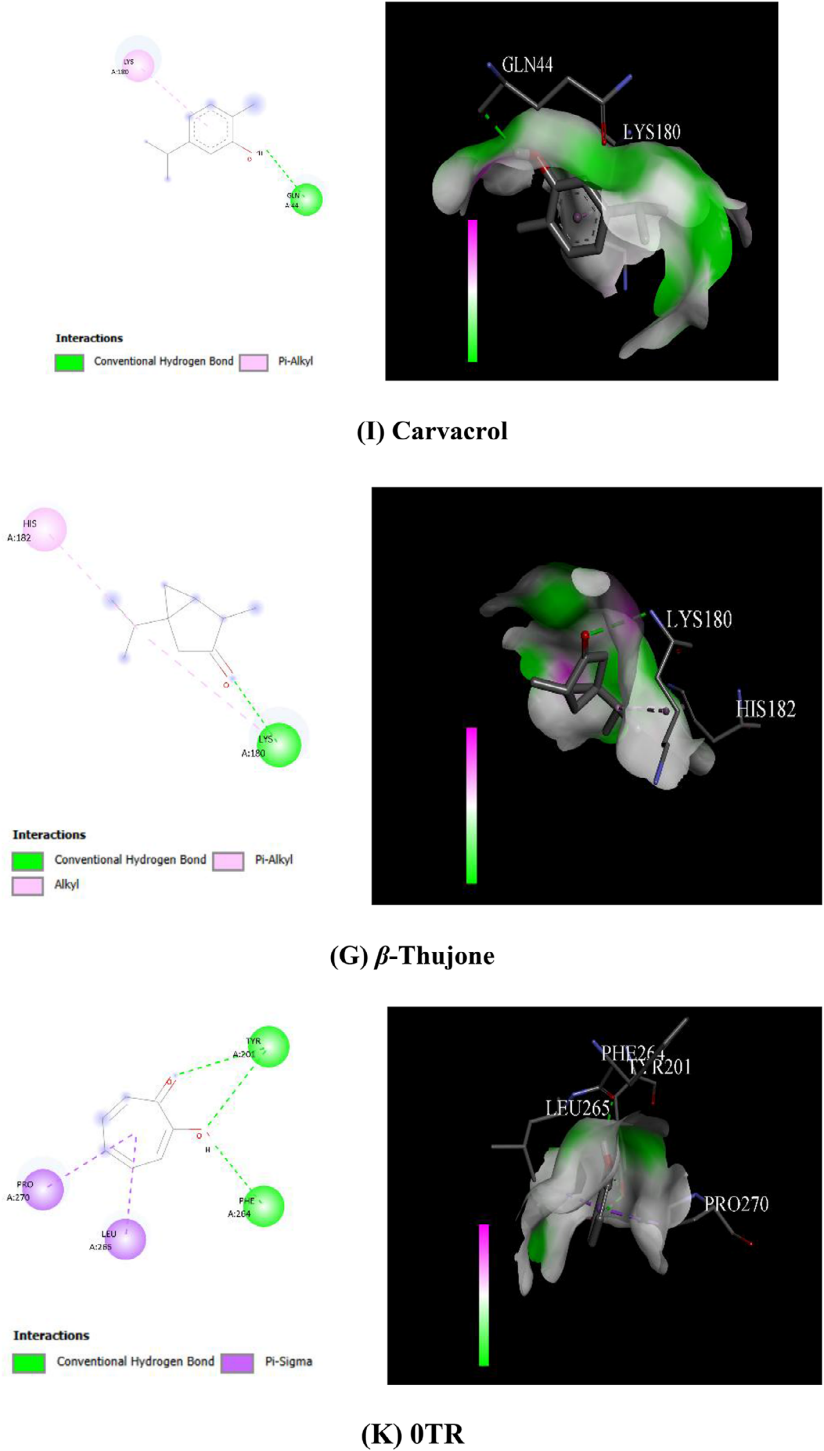

Among the volatile constituents, vanillin and kojic acid occupied a similar region within the active site. Vanillin interacted mainly through π–π stacking and π–alkyl contacts with **PHE264, HIS263, ALA286, and VAL283**, whereas kojic acid established both conventional hydrogen bonds and aromatic interactions involving residues of the catalytic pocket.

The interaction profiles of *α*‐thujone, carvacrol, *β*‐thujone, and the native ligand (0TR) were characterized by fewer favorable contacts and comparatively higher predicted binding energies. Since molecular docking provides a static representation of ligand–protein recognition and does not account for receptor flexibility, solvent effects, or binding kinetics, these interaction patterns should be regarded as qualitative structural predictions requiring experimental confirmation.

Overall, the docking analysis suggests that flavonoids, particularly quercetin, luteolin, and naringenin, exhibit the most favorable predicted interactions with tyrosinase among the investigated phytochemicals. Nevertheless, the relatively narrow range of docking scores indicates that these computational results should be interpreted as comparative estimates of ligand binding rather than definitive evidence of inhibitory potency. Therefore, the molecular docking analysis provides mechanistic support for potential ligand–target recognition but should not be directly correlated with the inhibitory activity or IC_50_ values obtained from the crude extracts.

#### 3.8.3 Limitations of the In Silico Predictions

Several limitations of the present in silico analysis should be acknowledged when interpreting the docking outcomes. First, AutoDock Vina relies on an empirical scoring function that approximates, rather than rigorously calculates, the Gibbs free energy of binding; predicted ΔG values should therefore be regarded as relative rankings rather than as quantitative dissociation constants. Second, the receptor was treated as a rigid body, so the docking does not capture protein‐side‐chain rearrangements upon ligand binding (induced‐fit effects), nor does it model the dynamic behavior of the dicopper catalytic center of tyrosinase; consequently, residues that coordinate the two Cu^2^^+^ ions are not explicitly handled, which limits the accuracy of predictions for metal‐directed inhibitors. Third, explicit water molecules were removed during receptor preparation and were not re‐introduced in the docking grid, so solvent‐mediated bridges and entropic penalties associated with water displacement are not represented. Fourth, ligand conformational sampling, although controlled by an exhaustiveness parameter, remains restricted relative to the full ensemble accessible in solution and may underestimate the flexibility of flavonoids such as quercetin and luteolin, which can adopt multiple rotameric states. Fifth, the docking was performed on mushroom (*Agaricus bisporus*) tyrosinase (PDB 2Y9X), and although the catalytic‐pocket architecture of mushroom and human tyrosinases shares several conserved histidine residues, the extrapolation of binding affinities to human melanogenesis should be made cautiously. Finally, the docking scores have not been validated by molecular dynamics simulations, MM‐GBSA/PBSA rescoring, or by direct measurement of the inhibitory constant of the isolated ligands against tyrosinase; therefore, the present in silico data are best viewed as a hypothesis‐generating tool that complements, rather than substitutes for, the in vitro assay.

## 4 Conclusion

This study provides a seasonal snapshot of the phytochemical profile and in vitro bioactivity of *A. herba‐alba* aerial parts harvested in the Aflou zone. Total phenolic (42.11–126.71 mg GAE·g^−^^1^ dw) and flavonoid (12.21–37.00 mg RE·g^−^^1^ dw) contents, essential oil yield (0.54%–0.71%), and chemical classes shifted markedly across the spring, summer, autumn, and winter collections, confirming that harvest date is a key variable for this species. The summer extract was the most potent phenolic‐rich fraction in the four antioxidant assays (DPPH IC_50_ = 29.40 ± 0.10 µg·mL^−^^1^; ABTS = 27.16 ± 0.07 µg·mL^−^^1^; FRAP = 0.436 ± 0.001 mmol·g^−^^1^) and the most potent tyrosinase inhibitor (IC_50_ = 2.89 ± 0.07 µg·mL^−^^1^), whereas the winter oil dominated by carvacrol (21.73%) showed the strongest activity among essential oils (DPPH IC_50_ = 67.79 ± 0.14 µg·mL^−^^1^; tyrosinase IC_50_ ± 45.70 ± 1.72 µg·mL^−^^1^). Molecular docking indicated that flavonoids, particularly quercetin, luteolin, and naringenin, exhibit the most favorable predicted interactions with tyrosinase among the investigated phytochemicals, consistent with their role as candidate inhibitors of melanin‐related enzymes. Nevertheless, the relatively narrow range of docking scores indicates that these computational results should be interpreted as comparative estimates of ligand binding rather than definitive evidence of inhibitory potency.

These observations are limited to chemical profiling and in vitro/in silico assays performed on one site and one growing year. No acute or chronic toxicity testing, no cell‐based melanogenesis assay, no skin permeation study, and no in vivo validation were carried out, so the present data should not be interpreted as evidence of therapeutic, nutraceutical, or cosmetic efficacy. Within those limits, the results may be useful as a preliminary guide for selecting summer material for phenolic‐rich extract preparation and winter material for phenolic‐monoterpene‐rich essential‐oil distillation, and as a starting point for the targeted phytochemical and toxicological studies that would be required before any applied use can be considered.

## Author Contributions

**Alia Djekidel**: performed the plant extraction, chemical experiments, data curation, and formal analysis. **Boulanouar Bakchiche**: designed and performed the experiments, interpreted the results, validation, data curation, visualization, drafted the manuscript, and revised it. **Hadjira Guenane**: performed supervision and investigation. **Imededdine Kadi**: helped in experimental work. **Hicham Gouzi**: funding acquisition. **Ilyas Yildiz**: conducted the chemical composition analysis. **Süleyman Muhammed Çelik**: conducted the chemical composition analysis. **Ramazan Erenler**: supervision; **Mosad A. Ghareeb**: designed and conceived the study, validation, data curation, visualization, wrote the manuscript, and revised it, supervision. **Stefania Garzoli**: writing – review and editing, supervision. All authors have read and agreed to the published version of the manuscript.

## Funding

This work was supported by Program (A16N01UN030120230001) from the university research‐ formation project of the General Directorate of Scientific Research and Technological Development that belongs to the Ministry of Higher Education and Scientific Research‐Algeria.

## Conflicts of Interest

The authors declare no conflicts of interest.

## Supporting information




**Supporting File 1**: cbdv71777‐sup‐0001‐SuppMat.docx.

## Data Availability

The data that support the findings of this study are available from the corresponding author upon reasonable request.
