# Supplementary material for: Seasonal Phytochemical Variation and Bioactivity of Artemisia herba‐alba: Comparative Evaluation of Essential Oils and Phenolic Extracts With Antioxidant, Tyrosinase Inhibitory and In Silico Studies
Source: Chem Biodivers. 2026 Sep 27;23(9):e71777. doi: 10.1002/cbdv.71777 (PMC13616480; doi:10.1002/cbdv.71777)
Supplement: Supplementary file 1 [file CBDV-23-e71777-s001.docx]

**Seasonal Phytochemical Variation and Bioactivity of *Artemisia herba-alba*: Comparative Evaluation of Essential Oils and Phenolic Extracts with Antioxidant, Tyrosinase Inhibitory and *In Silico* Studies**

**Alia Djekidel^1^, Boulanouar Bakchiche^1,2*^, Hadjira Guenane^1^, Imededdine Kadi^2^, Hicham Gouzi^1^, Ilyas Yildiz^3^, Süleyman M. Çelik^4^, Ramazan Erenler^5^, Mosad A. Ghareeb^6,*,#^, Stefania Garzoli^7,*#^**

^1^Laboratory of Biological and Agronomic Sciences (LBAS), Amar Telidji University, PB 37 G, 03000 Laghouat, Algeria. [a.djekidel.gpr@lagh-univ.dz](mailto:a.djekidel.gpr@lagh-univ.dz); [b.bakchiche@lagh-univ.dz](mailto:b.bakchiche@lagh-univ.dz); [ha.guenane@lagh-univ.dz](mailto:ha.guenane@lagh-univ.dz); [h.gouzi@lagh-univ.dz](mailto:h.gouzi@lagh-univ.dz);

^2^Research Unit of Medicinal Plants (RUMP), Laghouat 03000, Algeria. [kadi.imed.eddine@gmail.com](mailto:kadi.imed.eddine@gmail.com);

^3^Department of Molecular Biology and Genetics, Tokat Gaziosmanpasa University, Tokat 60000, Turkey. [lysyldz60@gmail.com](mailto:lysyldz60@gmail.com);

^4^Department of Medical Services and Techniques, School of Health Services, Igdir University, Igdir 76000, Turkey. [suleymancelik10@gmail.com](mailto:suleymancelik10@gmail.com);

^5^Department of Chemistry, Faculty of Arts and Sciences, Tokat Gaziosmanpasa University, Tokat 60240, Turkey. [rerenler@gmail.com](mailto:rerenler@gmail.com);

^6^Medicinal Chemistry Department, Theodor Bilharz Research Institute Kornaish El Nile, Warrak El-Hadar, Imbaba (P.O. 30), Giza 12411, Egypt. [m.ghareeb@tbri.gov.eg](mailto:m.ghareeb@tbri.gov.eg); mosad.ghareeb@tbri.sci.eg;

^7^Department of Chemistry and Technologies of Drug, Sapienza University, Rome, Italy

***Corresponding authors:** [stefania.garzoli@uniroma1.it](mailto:stefania.garzoli@uniroma1.it); [b.bakchiche@lagh-univ.dz](mailto:b.bakchiche@lagh-univ.dz); [m.ghareeb@tbri.gov.eg](mailto:m.ghareeb@tbri.gov.eg)

\# Mosad A. Ghareeb and Stefania Garzoli contributed equally to this work as last authors.

**Abstract**

The chemical composition and pharmaceutical properties of Artemisia herba-alba hydroalcoholic extract and essential oil have attracted substantial attention from researchers and the food industry. This study estimated the effect of seasonal variation on the chemical composition and biological activities of A. herba-alba extract and essential oil, focusing on antioxidant and tyrosinase inhibitory activities. Chemical profiling was achieved using LC–MS for the extract and GC–MS for the essential oil. Total phenolic and flavonoid contents were estimated by the Folin–Ciocalteu’s and aluminium chloride colorimetric assays. Antioxidant activity was evaluated using DPPH, ABTS, phosphomolybdate, and FRAP methods, while tyrosinase inhibition was expressed as IC₅₀ values using kojic acid as a positive control. Molecular docking was performed to explore the interactions of main constituents with the tyrosinase enzyme. The highest essential oil yield and phenolic and flavonoid contents were acquired in summer (0.71%, TPC 126.71 ± 0.34 mg GAE g⁻¹ dw; TFC 37.00 ± 0.55 mg RE g⁻¹ dw). LC–MS analysis identified naringenin, vanillin, luteolin, quercetin, and trans-cinnamic acid as the major compounds across all samples. GC–MS disclosed seasonal variation in essential oil composition; autumn oil contained several unique ingredients, remarkably davana ether 2 (41.15%), while winter oil was characterized by high carvacrol content (21.73%). The winter essential oil and summer extract exhibited the strongest antioxidant and tyrosinase inhibitory activities, with IC_50_ values of 45.70 ± 1.72 µg·mL⁻¹ and 3.06 ± 0.11 µg·mL⁻¹ for the oil and extract, respectively. Molecular docking revealed strong binding affinities of vanillin, kojic acid, and carvacrol within the catalytic pocket of tyrosinase, while thujone isomers exhibited weaker but consistent interactions.

**Keywords**: *Artemisia herba-alba*; Essential oils; Phenolics; Antioxidant; Tyrosinase inhibitory; Seasonal variation; *In Silico*; Health promotion

**Supplementary files:**

**Fig. S1.** Seasonal variation in total phenolic and total flavonoid contents.

*Bars represent mean ± SD (n = 3). Different letters indicate significantly different seasonal means according to Tukey’s HSD test (α = 0.05).*

**Fig. S2**. Total Ion Chromatogram (TIC) of the reference standard mixture (2000\ ppb) acquired on the Agilent 6460 Triple Quadrupole LC–MS/MS system

**Spring**

**Summer**

**Autumn**

**Winter**

**Fig. S3.** LC-MS chromatograms of the hydroalcoholic extracts of *A. herba-alba* collected in different seasons.

**Fig. S4.** GC-MS chromatogram of the essential oil of *A. herba-alba* collected in spring season.

**Fig. S5.** GC-MS chromatogram of the essential oil of *A. herba-alba* collected in summer season.

**Fig. S6.** GC-MS chromatogram of the essential oil of *A. herba-alba* collected in autumn season.

**Fig. S7.**  GC-MS chromatogram of the essential oil of *A. herba-alba* collected in winter season.

#### **Table S1**. Optimized LC–MS/MS acquisition and MRM parameters for target analytes.

| **Compound Name** | **Precersor Ion** | **Product Ion** | **Dwell** | **Fragmentor (V)** | **CE (V)** | **Polarity** |
| --- | --- | --- | --- | --- | --- | --- |
| Isoquercitrin | 464.9 | 302.8 | 9 | 95 | 8 | Positive |
| Polydatin | 391 | 229 | 9 | 80 | 8 | Positive |
| Epigallocatechin | 307 | 139 | 9 | 99 | 8 | Positive |
| Catechin | 291 | 165 | 9 | 80 | 12 | Positive |
| Catechin | 291 | 139 | 9 | 80 | 12 | Positive |
| Catechin | 291 | 123 | 9 | 80 | 12 | Positive |
| Sinapic acid | 224.9 | 206.9 | 9 | 90 | 3 | Positive |
| Sinapic acid | 224.9 | 174.8 | 9 | 90 | 3 | Positive |
| Syringic acid | 199 | 154.9 | 9 | 85 | 6 | Positive |
| Syringic acid | 199 | 140.2 | 9 | 85 | 6 | Positive |
| *trans*-ferulic acid | 195.1 | 176.9 | 9 | 75 | 10 | Positive |
| *trans*-ferulic acid | 195.1 | 144.7 | 9 | 75 | 10 | Positive |
| Vanillin | 152.9 | 124.9 | 9 | 65 | 13 | Positive |
| Vanillin | 152.9 | 93.1 | 9 | 65 | 13 | Positive |
| Vanillin | 152.9 | 65.2 | 9 | 65 | 13 | Positive |
| Rutin | 609.1 | 300.2 | 9 | 185 | 40 | Negative |
| Chlorogenic acid | 353 | 191 | 9 | 110 | 12 | Negative |
| Taxifolin | 303 | 284.8 | 9 | 144 | 6 | Negative |
| Taxifolin | 303 | 124.7 | 9 | 144 | 6 | Negative |
| Catechin | 288.9 | 245.1 | 9 | 140 | 12 | Negative |
| *trans*-ferulic acid | 193.1 | 133.9 | 9 | 95 | 14 | Negative |
| Caffeic Acid | 178.9 | 135 | 9 | 80 | 16 | Negative |
| Gallic acid | 168.9 | 124.9 | 9 | 100 | 12 | Negative |
| Vanillic acid | 167.1 | 152 | 9 | 96 | 12 | Negative |
| Vanillic acid | 167.1 | 123.1 | 9 | 96 | 12 | Negative |
| Vanillic acid | 167.1 | 107.7 | 9 | 96 | 12 | Negative |
| *p*-coumaric acid | 163.1 | 119.2 | 9 | 85 | 12 | Negative |
| Gentisic acid | 152.8 | 108.9 | 9 | 100 | 13 | Negative |
| Gentisic acid | 152.8 | 108 | 9 | 100 | 13 | Negative |
| Hydroxybenzaldehyde | 121 | 92 | 9 | 110 | 24 | Negative |
| Baicalin | 447 | 270.9 | 38 | 130 | 16 | Positive |
| Baicalin | 447 | 112.5 | 38 | 130 | 16 | Positive |
| Protocatechuic acid | 154.9 | 136.4 | 38 | 118 | 11 | Positive |
| Protocatechuic acid | 154.9 | 111 | 38 | 118 | 11 | Positive |
| Protocatechuic acid | 154.9 | 93.1 | 38 | 118 | 11 | Positive |
| Protocatehuic ethyl ester | 181 | 152.9 | 38 | 110 | 20 | Negative |
| Protocatehuic ethyl ester | 181 | 107.9 | 38 | 110 | 20 | Negative |
| *o*-coumaric acid | 162.8 | 119 | 38 | 66 | 13 | Negative |
| **Compound Name** | **Precersor Ion** | **Product Ion** | **Dwell** | **Fragmentor (V)** | **CE (V)** | **Polarity** |
| Protocatechuic acid | 152.9 | 108.9 | 38 | 150 | 14 | Negative |
| Salicylic acid | 137 | 93.1 | 38 | 85 | 18 | Negative |
| Morin | 302.9 | 136.8 | 18 | 145 | 32 | Positive |
| Morin | 302.8 | 153 | 18 | 145 | 30 | Positive |
| Quercetin | 302.8 | 152.9 | 18 | 125 | 34 | Positive |
| Quercetin | 302.8 | 136.9 | 18 | 125 | 34 | Positive |
| Kaempferol | 287 | 212.9 | 18 | 120 | 34 | Positive |
| Kaempferol | 287 | 164.7 | 18 | 120 | 34 | Positive |
| Kaempferol | 287 | 152.7 | 18 | 120 | 34 | Positive |
| Kaempferol | 287 | 120.9 | 18 | 120 | 34 | Positive |
| 7-Hydroxyflavone | 238.9 | 137.1 | 18 | 170 | 30 | Positive |
| Resveratrol | 229 | 135 | 18 | 105 | 14 | Positive |
| *trans*-cinnamic acid | 149.1 | 131.1 | 18 | 90 | 6 | Positive |
| *trans*-cinnamic acid | 149 | 131.1 | 18 | 75 | 6 | Positive |
| *trans*-cinnamic acid | 149 | 103 | 18 | 75 | 10 | Positive |
| Quercetin | 300.8 | 179 | 18 | 130 | 20 | Negative |
| Quercetin | 300.7 | 150.9 | 18 | 130 | 22 | Negative |
| Naringenin | 270.9 | 150.8 | 18 | 130 | 26 | Negative |
| Naringenin | 270.9 | 119.1 | 18 | 115 | 24 | Negative |
| Resveratrol | 227 | 184.9 | 18 | 130 | 22 | Negative |
| Resveratrol | 227 | 143 | 18 | 130 | 22 | Negative |
| *trans*-cinnamic acid | 147.1 | 103.1 | 18 | 70 | 8 | Negative |
| Diosgenin | 415.3 | 271.1 | 48 | 110 | 16 | Positive |
| Diosgenin | 415.3 | 253 | 48 | 110 | 16 | Positive |
| Luteolin | 285 | 133.1 | 48 | 132 | 36 | Positive |
| Biochanin A | 284.9 | 151.9 | 48 | 170 | 28 | Positive |
| Chrysin | 255 | 102.9 | 48 | 155 | 30 | Positive |
| Chrysin | 254.9 | 153 | 48 | 160 | 32 | Positive |
| 5-Hydroxyflavone | 238.9 | 165 | 48 | 170 | 36 | Positive |
| Biochanin A | 283 | 267.9 | 48 | 110 | 19 | Negative |

#### .

**Fig. S8.** DPPH radical-scavenging activity according to sample type and harvesting season.

*Bars represent mean ± SD (n = 3). Letters indicate Tukey HSD homogeneous subsets for the main effect of harvesting season (α = 0.05).*

**Fig. S9.** ABTS radical-scavenging activity according to sample type and harvesting season.

*Bars represent mean ± SD (n = 3). Letters indicate Tukey HSD homogeneous subsets for the main effect of harvesting season (α = 0.05).*

**Fig. S10.** Phosphomolybdenum (PM) antioxidant capacity according to sample type and harvesting season.

*Bars represent mean ± SD (n = 3). Letters indicate Tukey HSD homogeneous subsets for the main effect of harvesting season (α = 0.05).*

**Fig. S11.** Ferric reducing antioxidant power (FRAP) according to sample type and harvesting season.

*Bars represent mean ± SD (n = 3). Letters indicate Tukey HSD homogeneous subsets for the main effect of harvesting season (α = 0.05).*

**Fig. S12**. Seasonal dose–response curves for tyrosinase inhibition by *A. herba-alba* ethanolic extract.

**Fig. S13.** Tyrosinase inhibitory activity according to sample type and harvesting season.

*Bars represent mean ± SD (n = 3). Letters indicate Tukey HSD homogeneous subsets for the main effect of harvesting season (α = 0.05).*

**SPSS verification note:** All means, SDs, F-statistics, p-values, effect sizes, and Tukey seasonal groupings in this version were transcribed from the supplied SPSS output (resfhjj.pdf). For the two-way ANOVA, Tukey groupings reported by SPSS refer to the main effect of season (marginal means, n = 6 per season), not individual sample × season cells (n = 3). Accordingly, the letters on Figures 1–4 and 6 denote the season main-effect grouping.
